# Biomimetic Contact Behavior Inspired Tactile Sensing Array with Programmable Microdomes Pattern by Scalable and Consistent Fabrication

**DOI:** 10.1002/advs.202408082

**Published:** 2024-09-25

**Authors:** Xiaoliang Chen, Yizhuo Luo, Yun Chen, Sheng Li, Shizheng Deng, Bin Wang, Qi Zhang, Xiangmeng Li, Xiangming Li, Chunhui Wang, Juan He, Hongmiao Tian, Jinyou Shao

**Affiliations:** ^1^ Micro‐ and Nano‐technology Research Center, State Key Laboratory for Manufacturing Systems Engineering Xi'an Jiaotong University Xi'an Shaanxi 710049 China; ^2^ Frontier Institute of Science and Technology (FIST) Xi'an Jiaotong University Xi'an Shaanxi 710049 China; ^3^ Interdisciplinary Research Center of Frontier science and technology Xi'an Jiaotong University Xi'an Shaanxi 710049 China; ^4^ Shanxi Provincial Key Laboratory for Advanced Manufacturing Technology North University of China Taiyuan Shanxi 030051 China; ^5^ Department of Rehabilitation Medicine First Affiliated Hospital of Xi'an Jiaotong University Xi'an Shaanxi 710061 China

**Keywords:** embedded, flexible sensing array, multistage microstructure, octopus‐inspired, programmable

## Abstract

Flexible sensor arrays have attracted extensive attention in human‐computer interaction. However, realizing high‐performance sensor units with programmable properties, and expanding them to multi‐pixel flexible arrays to maintain high sensing consistency is still struggling. Inspired by the contact behavior of octopus antenna, this paper proposes a programmable multistage dome structure‐based flexible sensing array with robust sensing stability and high array consistency. The biomimetic multistage dome structure is pressurized to gradually contact the electrode to achieve high sensitivity and a large pressure range. By adjusting the arrangement of the multistage dome structure, the pressure range and sensitivity can be customized. More importantly, this biomimetic structure can be expanded to a multi‐pixel sensor array at the wafer level with high consistency through scalable and high‐precision imprinting technologies. In the imprinting process, the conductive layer is conformally embedded into the multistage dome structure to improve the stability (maintain stability over 22 000 cycles). In addition, the braced isolation structure is designed to effectively improve the anti‐crosstalk performance of the sensor array (crosstalk coefficient: 26.62 dB). Benefitting from the programmable structural design and high‐precision manufacturing process, the sensor array can be customized and is demonstrated to detect human musculation in medical rehabilitation applications.

## Graphical abstract

Inspired by the step‐by‐step contact principle of the octopus antenna interface, the programmable multistage dome structure sensor array is constructed to maintain high sensitivity in a wide sensitive range. Based on embedded silver nanowires, the sensor array can be manufactured in multi‐pixel with high consistency, and excellent sensing performance can be used in the field of human‐computer interaction.

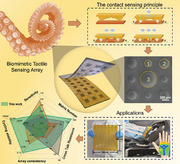

## 1 Introduction

With the progress in accurate measurement of pressure distribution in space, flexible pressure sensing arrays have attracted growing attention for potential applications in human‐computer interaction,^[^ ^1^, ^2^, ^3^, ^4^, ^5^ ^]^ image recognition,^[^ ^6^, ^7^, ^8^ ^]^ and wearable devices.^[^ ^9^, ^10^, ^11^, ^12^ ^]^ The system can be endowed with the perception ability owing to the sensor array, enabling it to detect the three‐dimension (3D) topography of the region accurately and extract useful information.^[^ ^13^, ^14^ ^]^ Part of pressure signals behave in large action areas, long action times, and irregular 3D regional morphology in practical applications.^[^ ^15^, ^16^ ^]^ However, due to the limitation of materials and manufacturing technology, the existing flexible pressure sensing arrays are difficult to manufacture in large areas and the arrays appear limited consistency.^[^ ^17^, ^18^, ^19^ ^]^ Secondly, the conductive layer is easy to fall off when serving for a long time, and the stability of the sensor array is also deficient.^[^ ^20^, ^21^ ^]^ In addition, when the acting area is uneven and the pressure size difference is large, how to achieve control of the sensitive interval while maintaining high sensitivity has received extensive attention.^[^ ^22^, ^23^ ^]^ Therefore, it is a challenge to develop a flexible pressure sensor array with a large detection area, high stability, high consistency, and adjustable sensing performance in the field of flexible electronics.

Resistive flexible pressure sensors have been extensively studied because of their simple structure, high sensitivity, strong anti‐interference ability, and easy implementation of the back‐end test system.^[^ ^24^, ^25^, ^26^ ^]^ Based on the permeation theory of composite materials, resistive flexible pressure sensors with simple processes have been developed.^[^ ^27^, ^28^, ^29^ ^]^ Nevertheless, the sensitivity of this sensing mechanism is low, and the array consistency is difficult to guarantee because the internal structure and components of the composite material are difficult to consistent during the manufacturing process. Using the interfacial contact resistance principle to fabricate resistive pressure sensors can significantly improve the sensitivity.^[^ ^30^, ^31^, ^32^ ^]^ The sandwich structure is a typical structure, and the sensor is composed of the microstructure layer, the conductive layer, and the electrode layer from top to bottom. The working principle of this sensing structure is achieved by changing the interface contact resistance between the conductive layer and the electrode layer. This structure can achieve sensitive sensing, but the conductive layer of the resistive sensor is usually manufactured by attaching, infiltrating, deposition, and sputtering processes,^[^ ^33^, ^34^, ^35^, ^36^ ^]^ which leads to the mismatch of Young's modulus between the microstructure and the conductive layer under pressure. The conductive layer of the sensor may be shed after a long time of operation, thus affecting the working stability of the sensor. Embedding materials such as silver nanowires and MXene in the sensor microstructure can improve the stability of the sensor.^[^ ^19^, ^20^ ^]^ Although some randomly sized protrusions created through the sandpaper, lotus leaf, and rose were reported previously to achieve a large linear range, their structure and performance were random and non‐reproducible, which strongly hamper the expansion and practical applications of the sensors.^[^ ^37^, ^38^, ^39^, ^40^ ^]^ Methods to fabricate the microstructure layer with a uniform structure such as lithography etching, laser direct writing, 3D printing, and others were also carried out to improve the consistency of the microstructure layer in a resistive flexible pressure sensing array.^[^ ^41^, ^42^, ^43^ ^]^ However, these performance control methods are often used on a single sensor unit, and it is still a challenge to manufacture complex structures consistently in multi‐pixel sensing arrays. The development of micro and nanostructure designs such as micro cones, hemispheres, micropillars, and micropores helps to achieve high sensitivity and wide operating range.^[^ ^44^, ^45^, ^46^, ^47^ ^]^ The combination of different unit structure compression conditions into step‐by‐step multistage interface contact has less attention while most research focuses on the unit structure design of microstructures.^[^ ^35^ ^]^ To control the increasing trend of interface contact area under pressure is the key to building a wide linear range pressure sensor array with high sensitivity. It is urgent to design and manufacture sensor arrays with large detection areas, high stability, high consistency, and wide range sensitivity to expand the application of sensor arrays in complex pressure environments.

Octopus tentacles are composed of suckers of different sizes and the small suckers can fill the gaps between the large suckers to achieve the maximum interface of octopus grasping touch.^[^ ^48^, ^49^, ^50^ ^]^ Inspired by this, in this study, by introducing the multistage dome structure as a conductive layer in contact with the electrode, we successfully constructed a novel resistive pressure sensor array with programmable control of linear range and sensitivity. Using the multistage dome structure to gradually contact the electrode surface under pressure, the linear sensitivity range of the sensor array is increased (0–100 kPa) under the condition of maintaining high sensitivity (0.15 kPa^−1^). The pressure range and sensitivity of the sensor can be customized by adjusting the arrangement and structural parameters of the multistage dome structure. The insertion of the silver nanowire conductive layer into the polydimethylsiloxane (PDMS) microstructure layer to form the embedded sensing layer solves the problem of the shedding of the conductive layer in long‐time use and achieves high cycle stability (22 000 times loaded at 15 kPa pressure). The sensor array was manufactured by a combination of spraying process and molding impression to achieve the consistency of the sensor array with high precision and large area manufacturing at the wafer level (calculated crosstalk coefficient CTK = 26.62 dB). In addition, the braced isolation structure designed on the surface of PDMS conductive film enables the sensor array to have high crosstalk resistance (array dispersion coefficient CV = 9.62%). The sensor can be customized to arbitrary size and shape according to the applications and can be used to detect pressure distribution such as gas pressure and human musculation to achieve human‐computer interaction and medical rehabilitation applications.

## 2 Results and Discussion

### 2.1 Inspiration and Sensing Mechanism of Multistage Dome Structure Sensor

**Figure 1:**
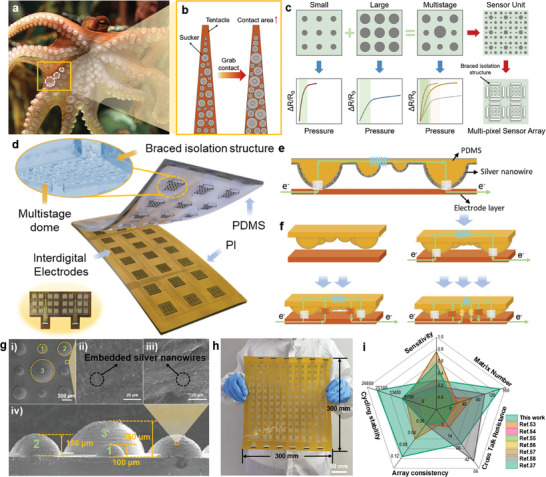
The sensing mechanism and performance of multistage dome structure sensor inspired by octopus. a,b) Sensing schematic diagram of octopus tentacles. c) Schematic diagram of dome microstructure combination. d) Schematic illustration of the embedded flexible pressure sensing array with multistage dome microstructures. e,f) Conduction principle of embedded multistage dome microstructures. g) Dimensional illustration and SEM images of multistage dome microstructures. h) Large format sensor array based on multistage structure. i) The performance of the proposed embedded flexible sensor array compared with other sensor arrays.^[^ ^37^, ^53^, ^54^, ^55^, ^56^, ^57^, ^58^ ^]^

Octopuses have a keen sense of touch and smell and can sense their surroundings through each tentacle.^[^ ^48^, ^51^ ^]^ The tentacles of an octopus are covered with suckers of varying sizes (**Figure** **1**a). When the tentacles bend or contact objects, different sizes of suckers can form the maximum contact surface to realize the haptic feedback (Figure 1b). Inspired by octopus tentacles, a sensing structure of programmable sensing contact surface based on multi‐size domes is proposed in this article. Dome structures of different sizes have different sensitivity and linear ranges, and combining multistage dome microstructures with different periods and diameters can improve the linear range while maintaining high sensitivity (Figure 1c). According to this principle, this article proposed a pressure‐sensing array based on the multistage dome microstructures (Figure 1d).

The conduction principle of the embedded multistage dome microstructures is illustrated in Figure 1e. Initially, only a few silver nanowires encountered the electrodes with slight pressures applied. As the pressure increases, the contact area between the large‐diameter domes and the electrodes increases, and the smaller domes begin to contact the cross‐electrodes at the same time (Figure 1f). Therefore, the multistage dome structure can maintain high sensitivity under small pressure and retain continuous growth of contact area under large pressure, consequently achieving a wide sensitive range. The equivalent circuit model of the flexible sensing unit is shown in Figure S1(Supporting Information). The sensing principle of the embedded flexible sensing unit is based on the theory of interface contact resistance change (Figures S2 and S3, Supporting Information). When the microstructure is stimulated by external pressure, the contact area with the electrode layer will change, resulting in a change in the total resistance R of the sensing unit.^[^ ^52^ ^]^ Therefore, the external pressure can be determined to achieve the purpose of sensing by measuring the resistance change of the sensing unit.

In this study, three‐stage composite dome structures with diameters of 200 µm, 300 µm, and 500 µm were fabricated and their scanning electron microscope (SEM) images are shown in Figure 1g. Figure 1g shows the multi‐size embedded dome structure prepared by molding with good structural consistency. The conductive material is embedded in the surface layer of the elastic dome microstructure, so the sensor exhibits high stability, high consistency, and high reliability. The composite structure of the three‐stage dome structure can increase the stress area of the sensor and disperse the stress to effectively reduce the pressure of single‐point overload and improve the reliability and accuracy of the sensor. In addition, the composite structure of the three‐stage dome structure can also improve the rigidity and stability of the sensor, while the traditional single‐layer dome structure is prone to plastic deformation, resulting in the loss of linear response of the sensor. The composite structure of the three‐stage dome structure can improve the rigidity and stability of the sensor while maintaining the flexibility of the conductive film, thus effectively broadening the linear interval of the sensor.

Based on the multistage dome structure, the programmable high‐performance sensing unit can be extended to multi‐pixel flexible arrays that maintain high sensing consistency and robust stability (Figure 1h). The embedded microstructure flexible pressure sensor array manufactured in this paper is compared with the sensors in other literature (Figure 1i; Table S1, Supporting Information).^[^ ^53^, ^54^, ^55^, ^56^, ^57^, ^58^ ^]^ It can be seen that: 1) Using the step‐by‐step contact sensing principle of multistage structure, the sensitivity of large pressure ranges can be improved. The biomimetic multistage dome structure is pressurized to gradually contact the electrode surface, breaking through the limitations of maintaining high sensitivity under large sensitive range; 2) The conductive material is embedded into the microdome structures in imprinting process to improve the stability and the high precision imprinting process can solve the problem of poor consistency in each unit in the large‐area expansion of multi‐pixel array; 3) The braced isolation structure effectively improves the crosstalk resistance performance of the sensor array. The braced isolation structure reduces the strain transfer between the sensor units, limiting the mechanical deformation caused by strain transfer, and thus inhibits the signal crosstalk phenomenon between the sensor arrays; 4) The manufacturing process of the flexible pressure sensor array that can be molded with multi‐pixel and high precision ensures the uniformity of the sensor unit performance and improves the consistency of the sensor array.

### 2.2 Manufacturing Process and Characterization of the Tactile Sensing Array

**Figure 2:**
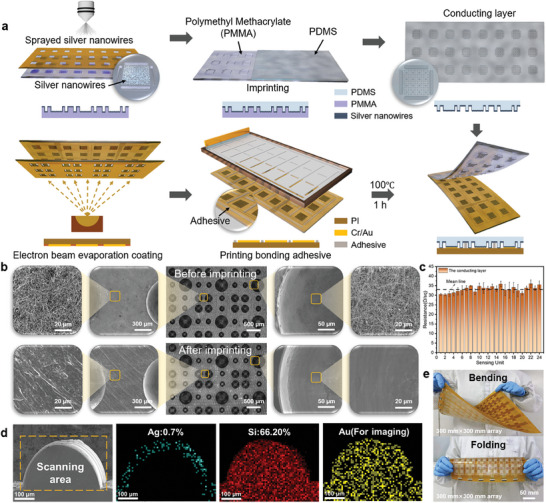
The manufacturing process and characterization of the tactile Sensing Array. a) The manufacturing process of the sensor arrays. b) SEM images of silver nanowires’ distribution on mold surface before and after imprinting. c) Square resistance distribution of silver nanowires in the conductive layer. d) Distribution of elements in dome microstructure. e) Flex and fold display of flexible pressure sensing arrays.

For sensor arrays, achieving high consistency of sensor unit sensitive structure and large area manufacturing is the premise of obtaining high precision performance of sensor arrays. The manufacturing process is shown in **Figure** **2**a. In this study, the conductive layer of silver nanowires was embedded into the microstructure layer of the PDMS dome by spraying and molding process, and the flexible pressure‐sensitive conductive film of the microstructure was controllably manufactured. The interdigital electrodes were manufactured by electron beam evaporation coating technology, and the pressure‐sensitive film and interdigital electrode were bonded and packaged by a hot pressing process. Thus, the pressure sensing array with high stability, high consistency, and high reliability is produced. The detailed steps for manufacturing are described in the section on materials and methods.^[^ ^59^ ^]^ SEM was used to observe the dome and interconnecting parts on the surface of the mold before and after imprinting (Figure 2b). It can be seen from the figure that after spraying, silver nanowires can be uniformly deposited on the surface of the mold. After imprinting, there is only a small amount of silver nanowires remaining on the surface of the connected part, and there are basically no silver nanowires remaining in the cavity of the mold dome. Compared with the SEM images before imprinting, it is obvious that the silver nanowires are basically taken away by PDMS curing, leaving only a trace amount of silver nanowires remaining on the surface of the mold. The results reveal the transfer of silver nanowires during the solidification of PDMS, and the silver nanowires can be successfully transferred into the PDMS structure during the imprinting process.

The spraying process used in the manufacturing process has high consistency and stability. After spraying, the square resistance of 24 sensing units was measured and the resistance distribution diagram was obtained as shown in Figure 2c and Figure S4 (Supporting Information). The resistance values of the conductive layer in the 24 sensing units are consistent, ranging from 6 to 9 Ω sq^−1^. Stability and durability under pressure loading and unloading conditions are important factors for flexible pressure sensing arrays to achieve high stability and durability. The silver nanowires embedded in a limited region with a low mass ratio do not destroy the high elasticity characteristic of PDMS material (Figure 2d; Figures S5 and S6, Supporting Information). Therefore, the conductive network of silver nanowires in the flexible pressure‐sensitive array conductive film has the same deformation resistance and recovery ability as the PDMS substrate under pressure loading and unloading conditions. The sensor array can be freely bent and folded and is suitable for a variety of complex environments and objects of different shapes (Figure 2e).

### 2.3 Sensing Properties of the Sensing Unit

**Figure 3:**
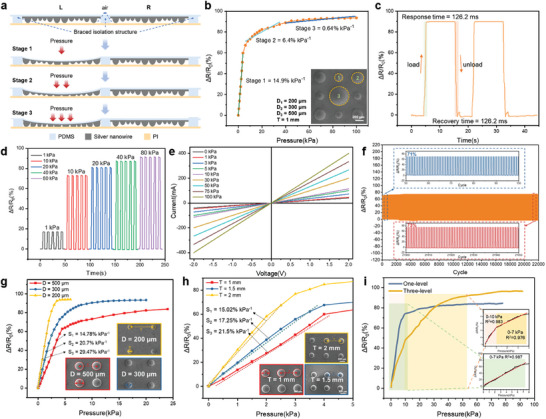
Sensing properties of the sensing unit. a) Schematic diagram of the braced isolation structure for anti‐crosstalk. b) Sensitivity curve of sensing unit. c) The response and recovery time of the sensing unit under 40 kPa pressure. d) The response curve of the sensing unit under different pressures. e) The I‐V curves of the sensing unit under various pressures. f) Cyclic stability test of the sensing array test for 22 000 repeat cycles under 15 kPa. g) Sensor pressure response tests for different dome diameters (D = 200 µm, D = 300 µm, D = 500 µm). h) Sensor pressure response testing for different array periods (T = 1 mm, T = 1.5 mm, T = 2 mm). i) Comparison of one‐level and three‐level composite dome structures.

A braced isolation structure is proposed in this study to effectively suppress signal crosstalk between sensor arrays (**Figure** **3**a). When external pressure is applied, the limiting effect of the braced isolation structure can effectively reduce the material deformation between adjacent sensing units, eliminating the effect of signal crosstalk. The three stages in Figure 3a correspond to the stages in Figure 3b. The pressure required for deformation at the pole of the dome is small, and the contact area between the dome structure and the electrodes increases rapidly with high sensitivity.^[^ ^60^, ^61^ ^]^ As the deformation area gradually approaches the equator of the dome, the required pressure is also increasing, and the growth rate of the contact area begins to slow down with decreasing sensitivity (Figure S7, Supporting Information). Therefore, the sensing unit presents different sensitivity in different pressure intervals (Figure 3b). The specific structural parameters and layout configurations of the tested sensor for Figure 3b–f are shown in Figure 1g and Experimental Section.

The sensor is designed and manufactured with high reliability and performance and can be used to achieve high‐precision pressure measurement and control. Short response recovery time is the prerequisite for the sensor to realize fast intelligent perception. The sensor unit responds stably to pressure loading and unloading, and the resistance change rate of the sensor unit is constant during the pressure maintenance stage (Figure 3c). Under the pressure of 40 kPa, the response time of the sensor when loading is 126.2 ms, and the recovery time when unloading is 131.5 ms. Pressure loads of 1, 2, 5, 10, and 20 kPa are applied to the sensing unit successively (Figure 3d). As the pressure increases, the resistance change rate of the sensing unit also increases, which indicates that the sensing unit is highly sensitive to pressure changes. In addition, during each pressure load cycle, the response of the sensing unit remains stable, indicating that the sensing unit has good pressure response characteristics and repeatability. The mechanical properties of the sensor are demonstrated in Figure S8 (Supporting Information). The connection strength of the sensor array is sufficient to maintain the pressure sensor serving for a long time without interface separation and the multistage dome structure has different compression characteristics under different strains (Figure S8, Supporting Information). The slope of the current–voltage(*I–V*) curve increases with the increase of the applied pressure in the range of 0–100 kPa pressure (Figure 3e), which proves that the resistance of the sensing unit decreases with the increase of the pressure. The I‐V curves show a good linear relationship under different pressures, which indicates that the microstructured flexible pressure sensor array has good ohmic characteristics and high resistance stability. The cyclic stability of the sensing array is tested for 22 000 repeat loading‐unloading cycles under 15 kPa (Figure 3f). The sensing performance has not significantly deviated after a long cycle test. The maximum resistance change rate of the initial response was 71% and the late response was 73%.

The sensitivity, pressure range, and linearity of the sensor can be programmed by adjusting the size and spacing of the multistage composite dome structure to meet the different requirements of different application scenarios. Three kinds of dome structures with different diameters were prepared (Figure 3g). The increase in dome diameter can increase the pressure limit of the sensor but reduce its sensitivity. The distance between the dome structures decreases when the diameter of the dome increases, resulting in the reduction of the pressure transmission range. The overall deformation resistance of the structure increases, and the pressure limit also increases. Under the same pressure change, the contact area rate of the large dome array structure is smaller than that of the small dome array. Therefore, the sensor sensitivity decreases with the increase of dome diameter. Three kinds of microstructure flexible pressure sensors with different array periods were prepared to investigate the effect of the array period on the performance of flexible pressure sensors (Figure 3h). The sensor with array period T = 1 mm has the smallest sensitivity S_1_ (0.15 kPa^−1^), while the sensitivity S_2_ of T = 1.5 mm is 0.17 kPa^−1^ and the sensor sensitivity S_3_ of T = 2 mm is 0.21 kPa^−1^. The increase in microstructure spacing will cause the sensor more prone to deformation, thus producing a larger pressure signal output. By adjusting the diameter of the dome and the period of the array, the sensitivity and measuring range of the sensor can be programmed (Figure S9, Supporting Information). More large‐size dome structures can be introduced into the multistage structure to obtain a larger linear range, while more small‐size dome structures can be added to the multistage structure to obtain higher sensitivity. In Figure S9 (Supporting Information), a finite element analysis using the simulation model was carried out as a bridge between theory and experiment and provided a theoretical basis and guidance for optimizing sensing performance. Figure 3i shows the comparison of one‐level and three‐level composite dome structures. The pressure sensor of the one‐level dome structure has high linearity in 0–7 kPa, and the correlation coefficient R^2^ is 0.987. The three‐level dome structure has high linearity within 0–10 kPa and 10–40 kPa, and its correlation coefficients R^2^ are 0.983 and 0.976 respectively. The pressure sensor of the three‐level composite dome structure can perform linear output within the pressure range of 0–40 kPa. Therefore, the three‐stage composite dome structure can widen the linear working interval of the sensor by nearly 6 times compared with the one‐level dome structure.

### 2.4 Performance and Application of the Sensor Arrays

**Figure 4:**
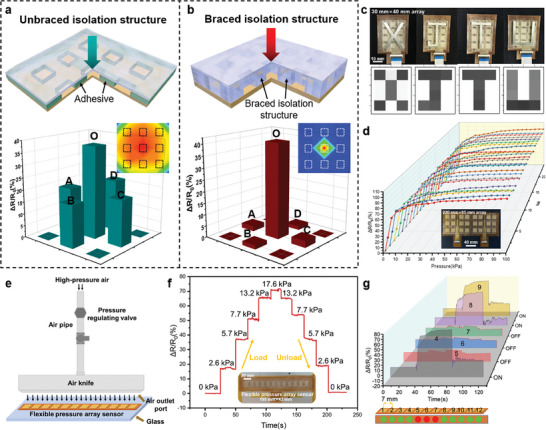
Performance and application of the sensor arrays. a,b) Comparison of signal crosstalk between unbraced isolation structure and braced isolation structure under 50 kPa pressure. c) Pressure image recognition of “XJTU” by the sensor array. d) Response curve of each sensor unit of 3 × 8 array pressure sensor. e) Schematic diagram of cleaning glass with an air knife. f) Air knife outlet flow pressure detection. g) Flexible pressure sensor array for air knife plugging location detection.

The braced isolation structure can effectively suppress signal crosstalk between sensor arrays (Figure 3a; Figure S10, Supporting Information). When external pressure *F* is applied to the working sensing unit, the limiting effect of the braced isolation structure can effectively reduce the material deformation between adjacent sensing units. As a result, material deformations between sensing units can be isolated, eliminating the effect of signal crosstalk. For the unbraced isolation structure, the voltage change rate of the stressed central sensing unit O is 82.06%, and the voltage change rate of the non‐stressed sensing units A, B, C, and D are 64.65%, 69.24%, 62.34%, and 59.24% respectively (Figure S11, Supporting Information). In this case, the crosstalk coefficient is 0.87 dB (**Figure** **4**a; Figure S11, Supporting Information). For braced isolation structure, the voltage change rate of the compression unit O is 85.38%, while the voltage change rate of the non‐stress units A, B, C, and D are 4.49%, 4.07%, 4.55%, and 4.89% respectively (Figure S11, Supporting Information). The crosstalk coefficient for the braced isolation structure is 26.62 dB, which is significantly lower than the sensor array without the braced isolation structure (Figure 4b; Figure S11, Supporting Information).

As shown in Figure 4c, the sensor array successfully recognizes the pressure generated by the four letters “XJTU” and its spatial distribution, indicating the sensor array's huge application prospect in the image recognition field. There are 24 pressure response curves in Figure 4d which respectively correspond to the 24 sensing units within the pressure range of 0–100 kPa. The pressure response of each sensing unit is basically the same without major deviation. The sensitivity of each sensing unit is relatively high within the pressure range of 0–10 kPa. The sensitivity begins to decrease because the resistance change rate of the sensing unit has reached more than 80% and tends to the saturation state with the pressure increase. When the pressure increases to 100 kPa, the resistance change rate of the sensing unit reaches >90% and the pressure range of the sensor reaches the limit state. The array dispersion coefficient CV is 9.62% < 10% (Figure S11, Supporting Information), which proves that the sensor array has high array consistency, ensuring the reliability and accuracy of the measurement.

An air knife is a high‐speed air injection equipment, which uses high‐pressure air to remove dirt, dust, grease, and other substances from the surface (Figure 4e). In the process of using the air knife, the air knife is easy to clog, resulting in reduced cleaning effect, reduced production efficiency, and even cannot work normally (Figure S12, Supporting Information). The microstructure flexible pressure array sensor can quickly and accurately detect the pressure change on the air knife, and locate the blockage point of the air knife in real time. The pressure regulator regulates the air pressure at the air knife inlet to a range of 0.3 to 0.7 MPa. Place the 1 × 12 sensor array under the air knife outlet to measure the response of the sensor array during the loading and unloading of the cleaning air. The sensor's response to the step change of air pressure loading and unloading is obvious, which verifies that the sensor can accurately detect the air pressure change of the air knife outlet (Figure 4f).

In this study, we controlled the air inlet pressure to 0.7 MPa and set blocking areas with lengths of 7, 14, and 21 mm at the air knife's air outlet to simulate the scene where the air knife is blocked when cleaning glass. The blockage point can be located according to the response of each sensing unit to the gas pressure. As shown in Figure S13 (Supporting Information), when the length of the blocked area is 7 mm, the pressure response of the single sensing unit under the blocked area is quite small. When the plugging length is extended to 14 mm, the two sensing units under the plugging area have no response. When the plugging length was further extended to 21 mm (Figure 4g), none of the three sensing units below the plugging area responded to pressure. Therefore, when the pressure response of a sensing unit is small, it can be considered that there is a blockage in the air knife outlet area corresponding to the sensing unit.

### 2.5 Combining Machine Learning Algorithm to Realize Medical Rehabilitation Application

Over the past 30 years, the incidence of stroke has continued to increase and is now a major health challenge for people around the world. Due to the damage to the central nervous system, some patients will have lateral limb motor dysfunction and lose the ability to handle fine movement, which seriously affects the quality of their life. In recent years, with the development of rehabilitation medicine and technology, more and more rehabilitation robots and human‐computer cross‐technology have been applied to the rehabilitation training of patients.^[^ ^62^ ^]^ Compared with passive rehabilitation training methods, active rehabilitation training can combine the exercise and rehabilitation training tasks of patients and turn the mechanical boring exercise into an active game by providing multi‐sensory feedback such as vision and hearing, so that patients with hemiplegia can enjoy rehabilitation training to achieve the ideal rehabilitation effect. Here, we conducted rehabilitation training on the palm musculation movement of patients through simulated flight games and provided real‐time feedback on the cloud image of palm musculation for patients. The random forest algorithm was used to classify the different operation actions of the patients to analyze the accuracy of the patients' actions and help the patients improve their training actions.

**Figure 5:**
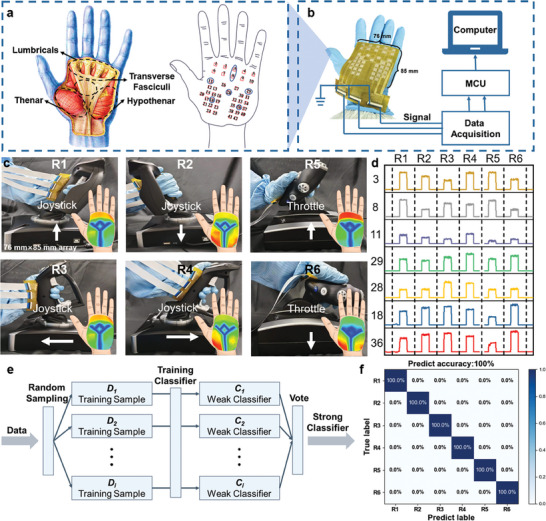
Combining machine learning algorithms to realize medical rehabilitation applications. a) The muscle distribution of the human palm and the setting of corresponding sensing units. b) Sensor signal processing diagram of sensor array. c) The muscle activities of the hand when controlling the throttle and the joystick. d) Pressure signals are collected by different sensing units during different operating actions. e) Classification process of random forest algorithm. f) Classification results of different operation actions.

The muscle distribution of the human palm is shown in **Figure** **5**a. To achieve a more accurate measurement, the sensor unit density of the palm sensor array is adjusted according to different parts and characteristics of the palm. The schematic of the signal processing of the sensor array for musculation detection is shown in Figure 5b. The test system consists of the palm sensor array, multi‐channel data acquisition, communication module, and host computer software. As shown in Figure S14 (Supporting Information), the sensor array is embedded in the glove to achieve stable sensing performance in the specific process. In the process of various palm movements, the sensor array can always be closely attached to the palm surface to maintain good contact between the sensor unit and the palm surface to achieve high‐precision measurement and reliability feedback.

Figure 5c shows the pressure cloud image of various muscle tissues of the patient's hand while controlling the throttle and joystick. Muscles have different force characteristics for different operational movements. Figure 5d shows the pressure signals collected by sensor units 3,8,11,29,28,18,36 (coded in Figure 5a) during six different operations. The four actions of pushing forward, pulling back, pushing left, and pushing right on the joystick are labeled R1‐R4 and the push forward and pull back on the throttle are labeled R5‐R6. Random forest algorithm combines several weak classifiers to get a strong classifier with remarkable classification performance, which has been proven to be an effective method for solving classification problems (Figure 5e). In this work, 180 sets of valid data were recorded and 30 times were randomly performed for each of the six actions. After preprocessing the data collected for each action, 70% of the data were randomly selected as the training set and the remaining data as the test set. The prediction accuracy of all data is up to 100%, which proves the effectiveness of the classification algorithm (Figure 5f). This method can distinguish between different hand movements by recognizing the muscle force of the human hand and can provide accurate feedback on the patient's fine motor accuracy of the hand, successfully realizing the application of medical rehabilitation.

Human tactile perception depends on the feedback of information from the nervous system. Similarly, the new generation of robots needs to integrate sensors with good sensing performance to process and feedback external information promptly. This tactile approach can be integrated into the new generation of robots to realize tactile perception. Moreover, the above experimental results provide important experimental data and theoretical support for further exploring the synergistic effect of hand muscles in the control process.

## 3 Conclusion

In summary, a programmable embedded flexible pressure sensor array based on multistage dome microstructures is proposed and successfully demonstrated. The sensor array can be scalable and manufactured by spraying and imprinting process. Through programming the multistage dome structure, the sensitivity is regulated between 0.15 and 0.29 kPa^−1^, the pressure limit is regulated between 10 and 100 kPa, and the linear working range is regulated from 0–7 kPa to 0–40 kPa, successfully realizing the control of the sensor array performance. The insertion of the silver nanowire conductive layer into the PDMS microstructure layer improves the stability of the sensor array, which can perform 22 000 cycles, ensuring a stable signal output in complex environments. In addition, the braced isolation structure between the sensor units reduces the calculated crosstalk coefficient CTK of the sensor array from 0.87 to 26.62 dB, greatly reducing the signal crosstalk between the rows of the sensor array. Finally, based on the scalability of the manufacturing process and the customization of the performance, the feasibility of the application of the sensor array in the field of human‐computer interaction and medical rehabilitation is verified. The sensor array has the characteristics of high stability, low crosstalk, high array consistency, and customizable performance, which can realize stable tactile perception in complex environments, and has wide application prospects in the field of electronic skin and medical rehabilitation.

## 4 Experimental Section

### Preparation of Conductive Film

The dome structure is used as a pressure‐sensitive unit and the design size of the dome structure is D = 500/300/200 µm in diameter, and the period of the dome array is T = 1 mm. The array molding die is designed as a 3 × 8 array. The acrylic material is processed by computer numerical control (CNC) milling, and a 12 mm × 12 mm sensing unit mold is prepared. The spacing distance between the sensing units is 12 mm. Horizontal spacing of 16 and 8 mm is set between the sensing units. A column structure of 10 mm × 2 mm × 200 µm is fabricated between the sensing units of the flexible pressure sensing array to avoid crosstalk between the rows of sensing signals. Polyimide film is used as the material for spraying the mask layer, and the required mask layer is manufactured by laser cutting. The spray mask layer is attached to the surface of the mold, and the silver nanowire solution is sprayed onto the surface of the molded impression mold through a grating path using an electric pressure spray gun. The spraying process can effectively deposit silver nanowires into the sensing unit (Figures 1g and 2b,c,d). Finally, the PDMS mixture was poured onto the surface of a molded imprinting die coated with a conductive silver nanowire layer and cured under pressure at 25 °C. After the PDMS pressure molding for 24 h, the PDMS was separated from the molding die to obtain a microstructured flexible pressure‐sensitive conductive film (Figure S15, Supporting Information).

### Fabrication of Flexible Array Electrode Layer

The flexible electrode layer consists of a 3 × 8 array of interdigital electrode units, each of which is 11 mm × 11 mm in size and consists of two pairs of interdigital fingers with a width of 2 mm, interdigital spacing of 1 mm, and the spacing between the two pairs of interdigital fingers is 500 µm. Some interdigital electrode units are spaced 16 mm horizontally, while the remaining units are spaced 8 mm horizontally to allow space for the lead layout of the array electrodes. A PI film with a thickness of 100 µm was selected and cut to a size of 220 mm × 85 mm as the base layer of the flexible array electrode to prepare the corresponding mask layer. Choose gold as the evaporation target to improve the conductivity of the interfinger electrode. In order to enhance the bonding force between gold and PI substrate, a layer of chromium film was steamed as the bonding layer before steaming gold film. The mask layer is fitted with a PI substrate, and the evaporation parameters are selected on the electron beam evaporation platform. The deposition thickness of chromium film is 20 nm and that of gold film is 150 nm. Finally, the PI base and mask were removed.

### Bonding Package of Sensing Arrays

The screen printing process is used to print bonding adhesives with a mesh number of 200 mesh. The four‐loop bonding width of the flexible array electrode is 6 mm, and the regional bonding width between the interdigital electrode units is 4 mm. The surface of the flexible array electrode was subjected to oxygen plasma bombardment treatment, and then the flexible array electrode was bonded with the screen‐printing plate. The PDMS bonding adhesive was configured according to the ratio of the PDMS matrix to curing agent 12:1. The bonding adhesive was then printed on the surface of the flexible array electrode using a scraper. After hydrophilic treatment, the conductive films were hot‐pressed and bonded with the flexible electrode layer. The heating procedure is at 100 °C for 60 min. Finally, the pressure is released to cool the molding.

### Finite Element Analysis

To study the compression characteristics of pyramidal, dome‐shaped, and cylindrical microstructures, commercial software ABAQUS 6.14 was used for numerical simulation analysis. Young's modulus of 1000 kg m^−3^ and Poisson's ratio of 0.45 were defined for the three microstructured pressure‐sensitive units, while the rigid plane was defined as a rigid body in its material properties. Load is applied to the upper surface of the rigid plane successively from 0 to 100 kPa with 5 kPa for each step to obtain the pressure visualization data of the three pressure‐sensitive microstructures.

### Measurements and Characterization

Field emission scanning electron microscopy (SU8010, HITACHI) and laser scanning confocal microscopy (OLS4000, Olympus) were used to characterize the structure of the multistage dome with an embedded conductive layer. The sensing unit of the flexible pressure sensor array is fixed on the load platform of the pressure gauge (PT‐1198), and a soft block is attached to the pressure gauge sensor press head so that the pressure generated by the pressure gauge can be uniformly transferred to the surface of the sensor. The pressure is slowly loaded on the surface of the sensor unit. The pressure sensing performance of the sensor array is tested by measuring the resistance change through the source meter (B2912A, Keysight). According to Ohm's law, the voltage value curve is transformed into a resistance value curve, and the pressure rise stage of the pressure curve of the press, and the resistance value curve of the sensing unit are analyzed and obtained. The pressure data are interpolated to calculate the initial resistance of the sensor and the resistance change after pressure, and the resistance change rate‐pressure curve of the sensing unit is obtained.

## Conflict of Interest

The authors declare no conflict of interest.

## Author Contributions

X.C. and Y.L. contributed equally to this work. X.C., Y.L., and H.T. conceptualized the idea and designed the experiments. H.T. and J.S. supervised the project. X.C. and Y.L. prepared the sensor array with embedded multistage microstructures and realized its application. Y.C. and Q.Z. prepared the one‐lever structure samples and tested their performances. Y.L. and Y.C. characterized and analyzed the structure of samples. X.C., Y.L., Y.C., S.L., S.D., B.W., X.L., X.L., H.T., and J.H. analyzed and interpreted the data. All authors commented on the experimental results. X.C., Y.L., and J.S. co‐wrote the manuscript.

## Supporting information



Supporting Information

## Data Availability

The data that support the findings of this study are available from the corresponding author upon reasonable request.
