# Supplementary material for: Biomimetic Contact Behavior Inspired Tactile Sensing Array with Programmable Microdomes Pattern by Scalable and Consistent Fabrication
Source: Adv Sci (Weinh). 2024 Sep 25;11(43):2408082. doi: 10.1002/advs.202408082 (PMC11578381; doi:10.1002/advs.202408082)
Supplement: Supplementary file 1 — Supporting Information [file ADVS-11-2408082-s001.docx]

Supporting Information

Biomimetic Contact Behavior Inspired Tactile Sensing Array with Programmable Microdomes Pattern by Scalable and Consistent Fabrication

Xiaoliang Chen^‡^, Yizhuo Luo^‡^, Yun Chen, Sheng Li, Shizheng Deng, Bin Wang, Qi Zhang, Xiangmeng Li, Xiangming Li, Chunhui Wang, Juan He, Hongmiao Tian*, Jinyou Shao*

‡ These authors contributed equally to this work.

***Corresponding author’s e-mail: hmtian@xjtu.edu.cn**; **jyshao@mail.xjtu.edu.cn**

Supplementary Figure Captions:

***Figure S1****:* The equivalent circuit model of the flexible sensing unit.

***Figure S2****:* The sensing principle of the mosaic flexible sensing unit.

***Figure S3****:* Simulation of compression characteristics of three different microstructures.

***Figure S4****:* Square resistance distribution in the sensing area.

***Figure S5****:* Analysis of elements types in micro-dome structure by energy dispersive spectrometer.

***Figure S6****:* Distribution of elements in micro-dome structure.

***Figure S7****:* Compressive hyperelastic theoretical model of dome structure.

***Figure S8****:* Mechanical properties of sensor arrays.

***Figure S9****:* Finite element analysis of dome structures with different dimensions and configurations.

***Figure S10****:* Signal crosstalk test.

***Figure S11****:* Diagram of braced isolation structure and unbraced isolation structure.

***Figure S12****:* The air speed and pressure loss of air knife under different plugging area lengths.

***Figure S13****:* Blockage location detection for air knives.

***Figure S14****:* Custom manufactured flexible pressure sensing array based on human palm muscle distribution.

***Figure S15****:* Cloud image of pressure sensitive film thickness.

***Table S1****:* Comparison between this work and previously reported pressure sensors.


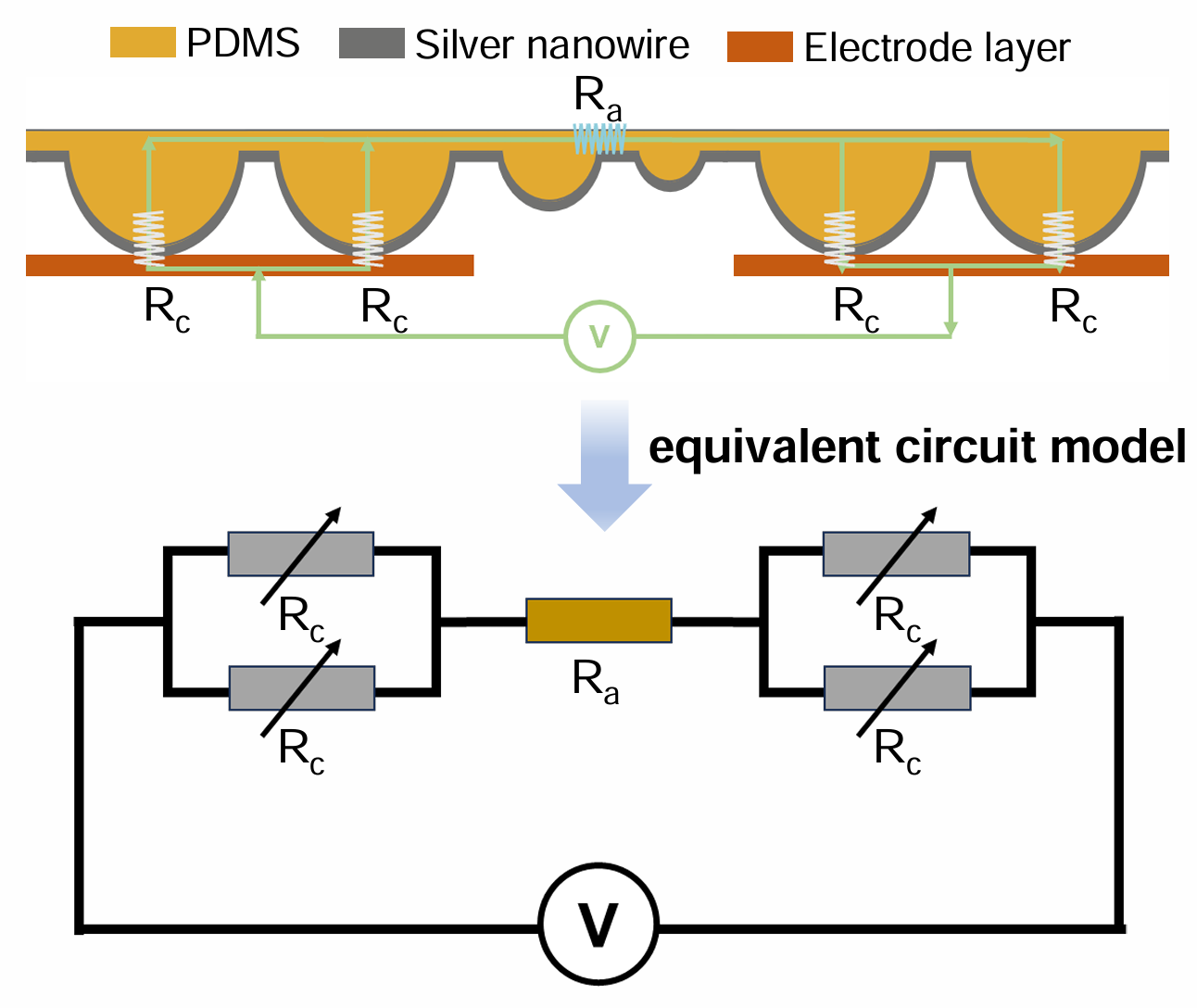


**Figure S1. The equivalent circuit model of the flexible sensing unit.**

The total resistance *R* of the sensing unit is mainly composed of the contact resistance *R_c_* between the interdigital electrode and the microstructure and the interconnected film resistance *R_a_* between the microstructure array. The contact resistance *R_c_* changes with the change of the contact area and is regarded as a variable resistance. The film resistance *R_a_* changes very little under pressure, so it can be regarded as a fixed resistance. The interdigital electrode has extremely high electrical conductivity, and its resistance can be ignored. In conclusion, the entire resistance model can be equivalent to several variable contact resistors in parallel and then connected in series with a fixed film resistance. The total resistance *R* of the sensing unit can be expressed as:

$$\begin{aligned} \text{R=}\frac{\text{R}_{\text{c}}}{\text{2}}\text{+}\text{R}_{\text{a}}\text{+}\frac{\text{R}_{\text{c}}}{\text{2}}\text{=}\text{R}_{\text{c}}\text{+}\text{R}_{\text{a}}\#\left( S1. \right) \end{aligned}$$

*R_c_* is the contact resistance, *R_a_* is the resistance of the film.

From the resistance law, the film resistance *R_a_* can be expressed as:

$$\begin{aligned} \text{R}_{\text{a }}\text{=}\text{ }\text{ρ}_{\text{a}}\frac{\text{L}}{\text{S}}\#\left( S2. \right) \end{aligned}$$

where $\text{ρ}_{\text{a}}$ is the surface resistivity of the film, *L* is the thickness of the conductive film between adjacent electrodes, and *S* is the cross-sectional area of the conductive film between adjacent electrodes.

The film resistance *R_c_* can be expressed as:

$$\begin{aligned} \text{R}_{\text{c}}\text{ = }\frac{\text{ρ}_{\text{c}}}{\text{A}}\#\left( S3. \right) \end{aligned}$$

where $\text{ρ}_{\text{c}}$ is the conductivity of the array microstructure at the contact point of the interdigital electrode, and *A* is the contact area between the array microstructure and the interdigital electrode. The total resistance *R* of the sensing unit can be expressed as:

$$\begin{aligned} \text{R = }\text{R}_{\text{c}}\text{ + }\text{R}_{\text{a }}\text{= }\frac{\text{ρ}_{\text{c}}}{\text{A}}\text{ + }\text{ρ}_{\text{a}}\text{ }\frac{\text{L}}{\text{S}}\#\left( S4. \right) \end{aligned}$$

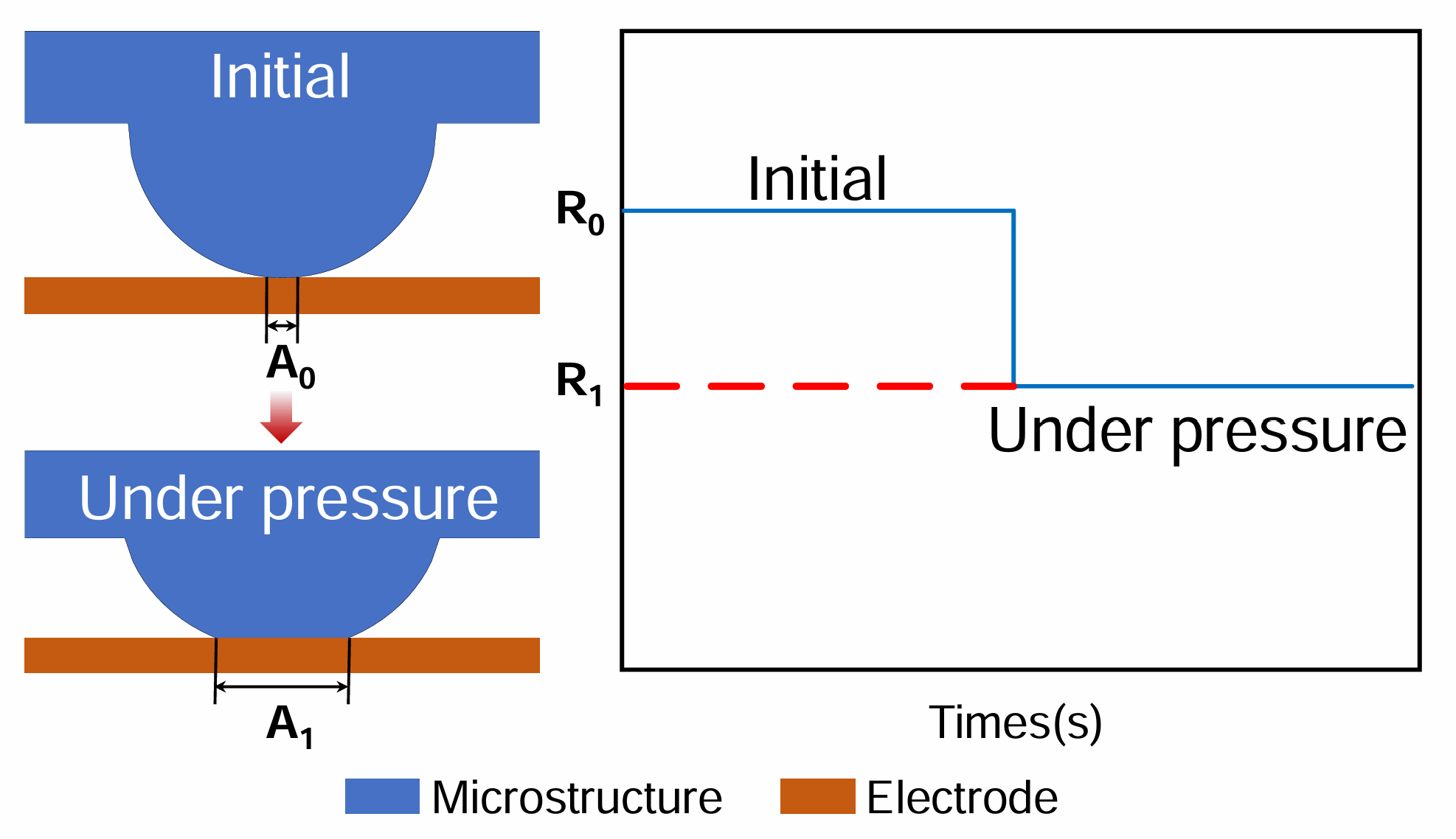


**Figure S2. The sensing principle of the mosaic flexible sensing unit.** According to the equation S4, the contact area *A* is inversely proportional to the resistance *R* of the sensing unit. When the contact area increases from *A_0_* to *A_1_*, the total resistance of the sensing unit also decreases from *R_0_* in the initial state to *R_1_* in the pressure state.


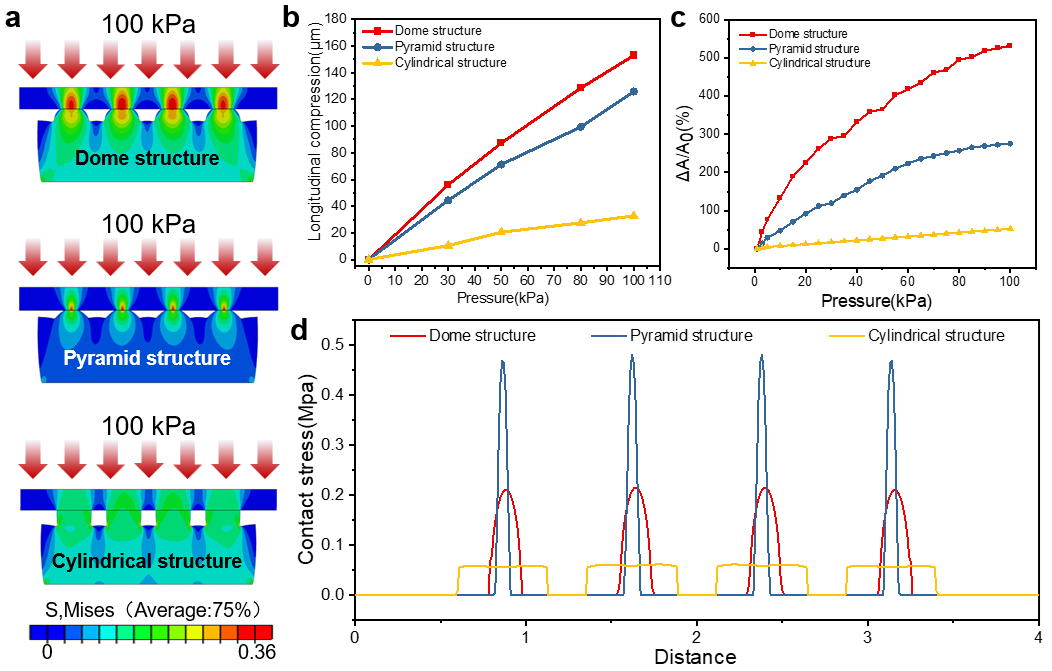


**Figure S3. Simulation of compression characteristics of three different microstructures.** (a) Stress conditions of three different microstructures under 100 kPa. The deformation occurs obviously when the pyramid and dome structure are subjected to external pressure, while the cylindrical structure is not obvious under the stimulation of external pressure. (b) Relationship between axial compression and pressure load of three microstructures. The compression of dome structure and pyramid structure is significantly higher than that of cylinder structure. (c) Relationship between compression contact area and pressure load of three kinds of microstructures. The change rate of the contact area of the dome structure is large, and the contact area is still increasing steadily when the contact area of the pyramid structure begins to reach the saturation state, indicating that the dome structure has better sensitivity and higher bearing capacity. (d) Stress distribution of three microstructures under pressure. The stress will accumulate at the tip of the dome structure and pyramid structure when the external pressure is applied. This concentrated stress will easily cause the structure to deform, so that the shape variable of the dome structure and pyramid structure is much larger than that of the cylindrical structure under the same pressure load.


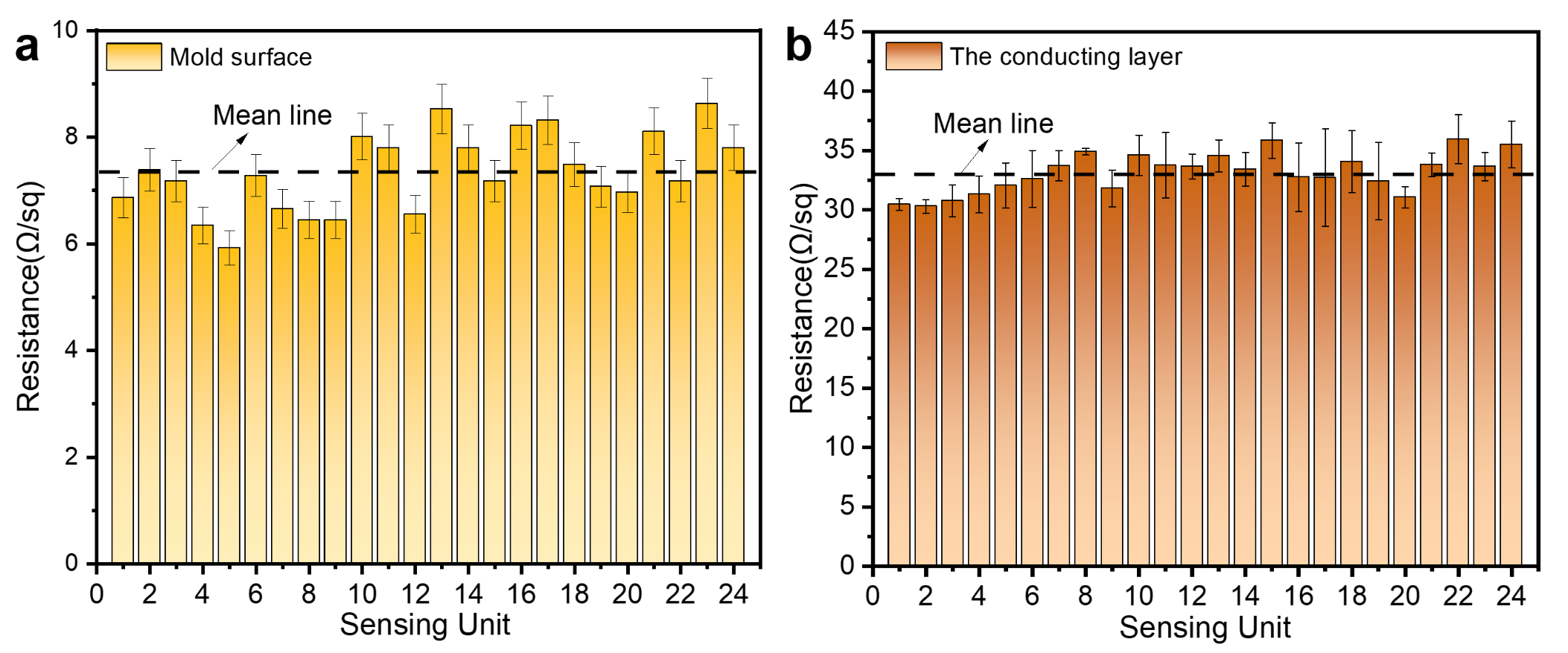


**Figure S4. Square resistance distribution in the sensing area.** (a) Square resistance of the 24 sensing units on the surface of the die after spraying. The spraying process can effectively deposit silver nanowires into the sensing units with high consistency and stability. (b) Square resistance of 24 sensing units of the conducting layer after embossing.

**Figure S5. Analysis of elements types in micro-dome structure by energy dispersive spectrometer.** C, O and Si are the constituent elements of PDMS material, and Ag represents silver nanowires inside the dome structure. Au element is sprayed on the surface of the micro-dome structure to meet the imaging needs of EDS spectrometer.


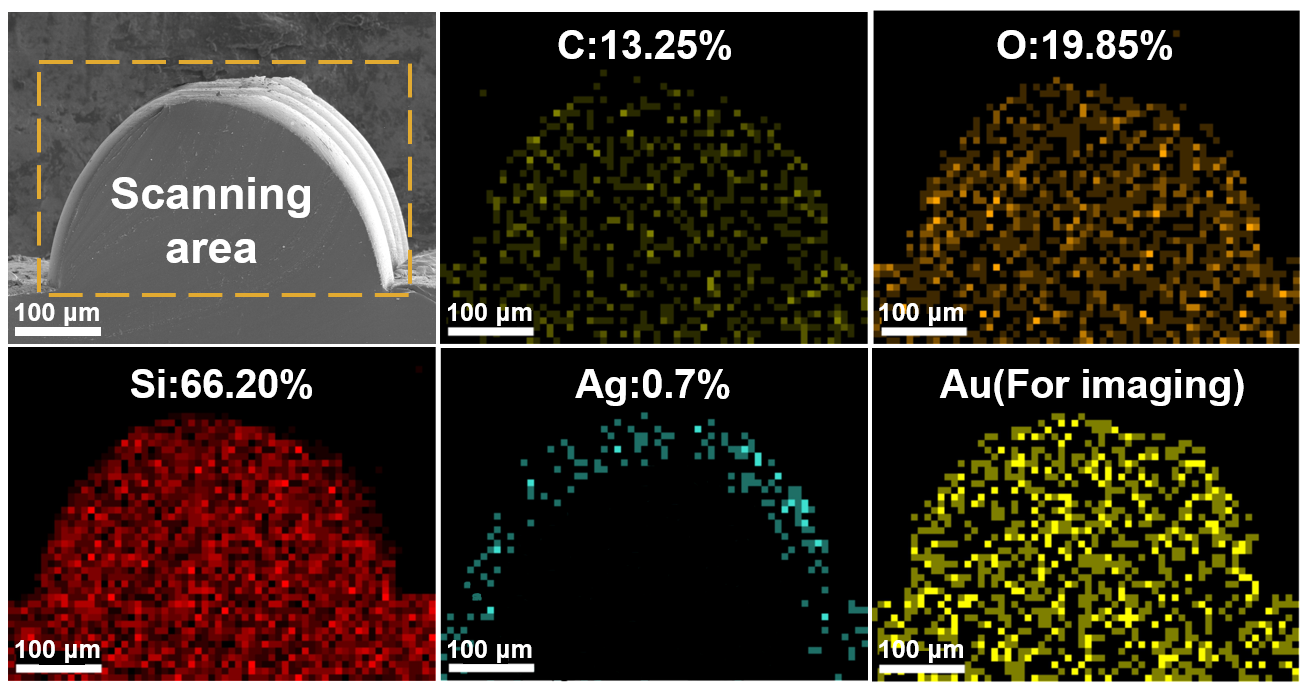


**Figure S6. Distribution of elements in micro-dome structure.** The proportion of silver element particles in the whole dome structure is only 0.7%, and silver nanowires are only embedded in a limited area on the upper surface of the PDMS dome structure.


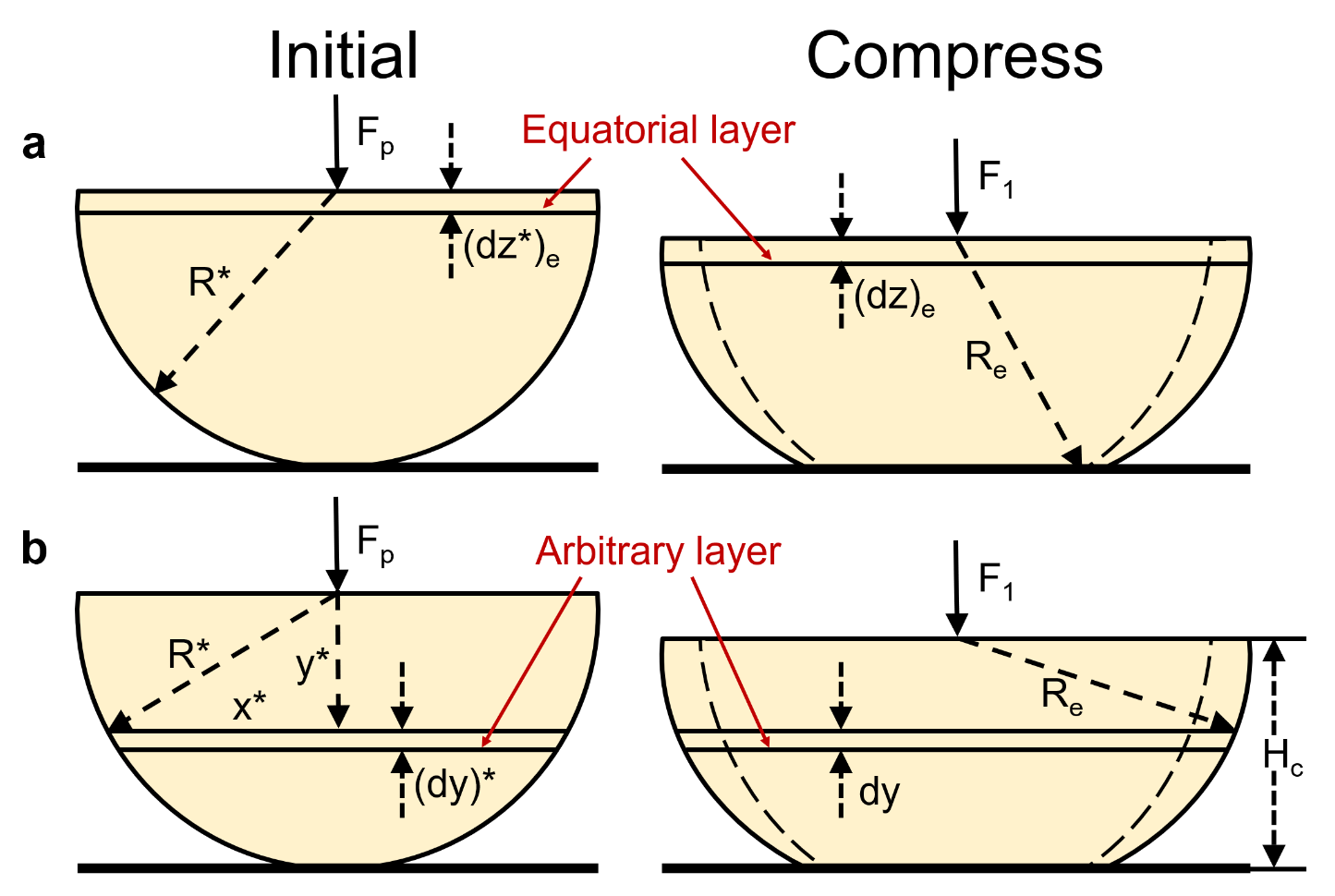


**Figure S7. Compressive hyperelastic theoretical model of dome structure.** (a) Schematic diagram of equatorial layer compression of dome structure. (b) Schematic diagram of arbitrary layer compression of dome structure.

The spherical neo-Hookean model is adopted to divide the entire hemisphere into infinite layers along the Y-axis. Based on the principle of calculus, the equatorial layer is regarded as a micro-cylinder with diameter *R** and thickness *(dz*)_e_*, which will become a flatter but wider micro-cylinder with radius *R_e_* and thickness *(dz)_e_* after compression. Dome structure of compressive stress $\sigma_{e}$ can be defined as:

$$\begin{aligned} \text{σ}_{\text{e}}\text{=}\frac{\text{F}}{\text{π}\text{R}^{\text{*2}}}\#\left( S5. \right) \end{aligned}$$

The compression strain ratio $\text{α}_{\text{e}}$ of the equatorial layer is the ratio of the thickness of the microcylinder before and after compression:

$$\begin{aligned} \text{α}_{\text{e}}\text{=}\frac{\text{(}\text{dz}\text{)}_{\text{e}}}{\text{(}\text{d}\text{z}^{\text{*}}\text{)}_{\text{e}}}\#\left( S6. \right) \end{aligned}$$

For incompressible models, the volume remains unchanged before and after compression:

$$\begin{aligned} \text{π}\text{R}^{\text{*2}}\text{·(}\text{d}\text{z}^{\text{*}}\text{)}_{\text{e}}\text{=π(}\text{R}_{\text{e}}\text{)}^{\text{2}}\text{·(}\text{dz}\text{)}_{\text{e}}\#\left( S7. \right) \end{aligned}$$

According to equation S6, the formula of compressive strain ratio $\text{α}_{\text{e}}$ at the equator can be rewritten as:

$$\begin{aligned} \text{α}_{\text{e}}\text{=(}\frac{\text{R}^{\text{*}}}{\text{R}_{\text{e}}}\text{)}^{\text{2}}\#\left( S8. \right) \end{aligned}$$

As shown in Fig. S4(b), if the vertical distance from the center of the sphere is *y** and the thickness is the same as that of the equatorial layer, the radius *x** of the layer is taken as follows:

$$\begin{aligned} \text{y}^{\text{*2}}\text{=}\text{R}^{\text{*2}}\text{-}\text{x}^{\text{*2}}\#\left( S9. \right) \end{aligned}$$

The compression height *H_c_* can be expressed as:

$$\begin{aligned} \text{H}_{\text{c}}\text{=}\int_{\text{0}}^{\text{h}_{\text{max}}} \text{1}\text{d}\text{y}\text{=}\int_{\text{0}}^{\text{R}^{\text{*}}} \text{α}\text{d}\text{y}^{\text{*}}\#\left( S10. \right) \end{aligned}$$

Where α is the local compression strain ratio.

By normalizing the deformation of the dome structure in the *x* direction relative to the *y* direction, we can get:

$$\begin{aligned} \beta=\int_{0}^{1} \text{α}_{\text{ϕ}}\text{d}\text{ϕ}\#\left( S11. \right) \end{aligned}$$

Here,

$$\begin{aligned} \text{β}\text{=}\frac{\text{R}_{\text{c}}}{\text{R}^{\text{*}}}\text{,}\text{ϕ}\text{=}\frac{\text{y}^{\text{*}}}{\text{R}^{\text{*}}}\#\left( S12. \right) \end{aligned}$$

$\text{R}_{\text{c}}$ is the compression radius of the contact area, and $\text{y}^{\text{*}}$ is the vertical distance from the compressed layer to the center of the sphere.

With the $\text{ϕ}$ ranging from 0 at the center of the sphere to 1 at the apex, the normalized dimensionless stress $\text{Q}_{\text{e}}$ is defined as:

$$\begin{aligned} \text{Q}_{\text{e}}\text{=}\frac{\text{σ}_{\text{e}}}{\text{G}}\text{=}\text{α}^{\text{-2}}\text{-}\text{α}\#\left( S13. \right) \end{aligned}$$

$\text{G}$ is the shear modulus of the material.

The local stress for any compressed layer to $\text{ϕ}$ can be expressed as:

$$\begin{aligned} \text{σ}_{\text{ϕ}}\text{=}\frac{\text{f}}{\text{π}\text{x}^{\text{*2}}}\text{=}\text{G}{\text{(}\text{α}^{\text{-2}}\text{-}\text{α}\text{)}}_{\text{ϕ}}\#\left( S14. \right) \end{aligned}$$

$\text{x}^{\text{*}}$ is the radius of any compressed layer.

In combination with equation S10, for a compressed layer of any nominal height, the rate of change of the dimensionless stress with respect to the equatorial layer can be expressed as:

$$\begin{aligned} \frac{\text{σ}_{\text{ϕ}}}{\text{G}}\text{=}{\text{(}\text{α}^{\text{-2}}\text{-}\text{α}\text{)}}_{\text{ϕ}}\#\left( S15. \right) \end{aligned}$$

Thus, we can calculate the strain ratio $\text{ϕ}_{\text{c}}$ at nominal height. Combining equations S9, S10 and S15, we get the compression radius $\text{R}_{\text{c}}$ in the contact area and the height variation $\Delta\text{h}$ in the current compression state:

$$\begin{aligned} \text{R}_{\text{c}}\text{=}\text{R}^{\text{*}}\text{×}\sqrt{\frac{\text{1-}\text{ϕ}_{\text{c}}^{\text{2}}}{\text{α}_{\text{c}}}}\#\left( S16. \right) \end{aligned}$$

$$\begin{aligned} \text{Δ}\text{h}\text{=(1-}\text{β}\text{)}\text{⋅}\text{R}^{\text{*}}\#\left( S17. \right) \end{aligned}$$

$\text{R}^{\text{*}}$ is the initial radius of the dome structure, $\text{ϕ}_{c}$ is the nominal height of the contact area, $\text{α}_{\text{c}}$ is the tangential principal elongation of the contact area, and $\text{β}$ is the total height compression ratio.


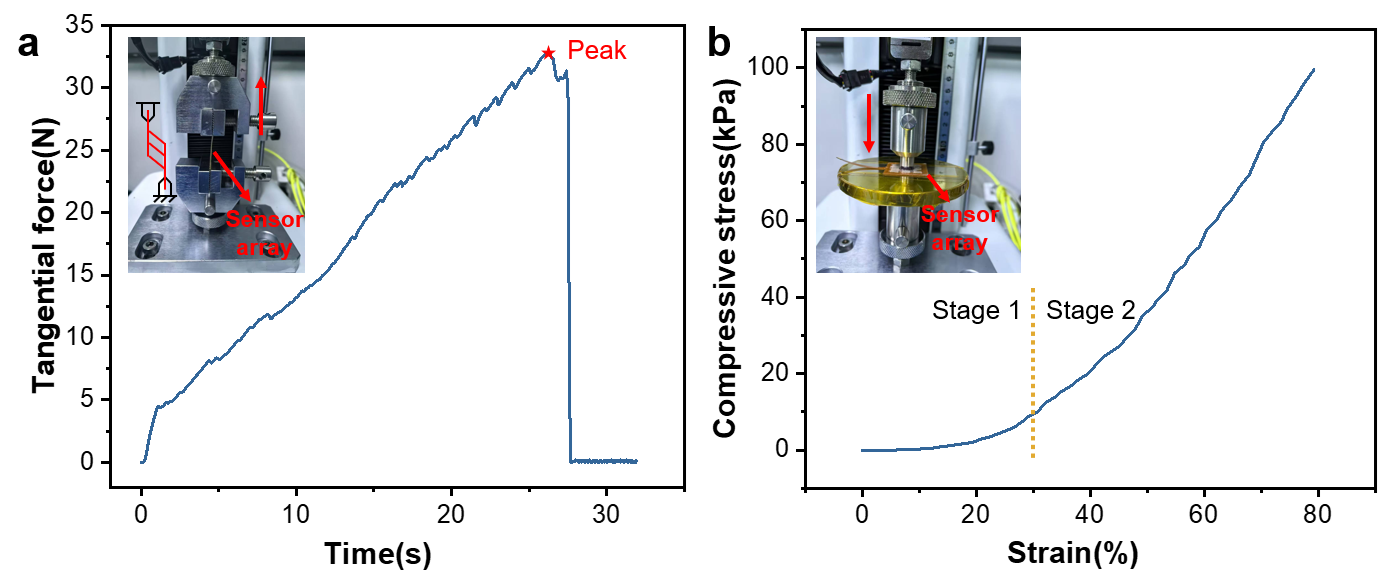


**Figure S8. Mechanical properties of sensor arrays.** (a) Connection strength of the sensor array. The effective area of PDMS connection is about 1 cm^2^ and the maximum tangential force the device can withstand is 32.8 N. The maximum shear strength after calculation is about 328 kPa, which far exceeds the working pressure range of the sensor. (b) Stress–strain curve of the sensor under compression. Stress is applied to the sensing unit using a cylindrical rod with 1 cm basal diameter and the sensor was compressed at a compression rate of 50 mm/min. There are two different deformation stages in the compression process: as the compressive strain increases, the stress increases slowly when the strain is less than 30% (stage 1), and after that, it increases sharply (stage 2). This phenomenon is mainly attributed to the structural changes of the dome structure under pressure. In stage 1, the deformation of some large domes plays a dominant role in the increase of compressive strain, resulting in large deformation and low stress (from 0 kPa to 10.05 kPa). In stage 2, the number of dome structures under pressure increases gradually until all the dome structures are under pressure, and the compression resistance increases significantly, reaching 99.52 kPa.


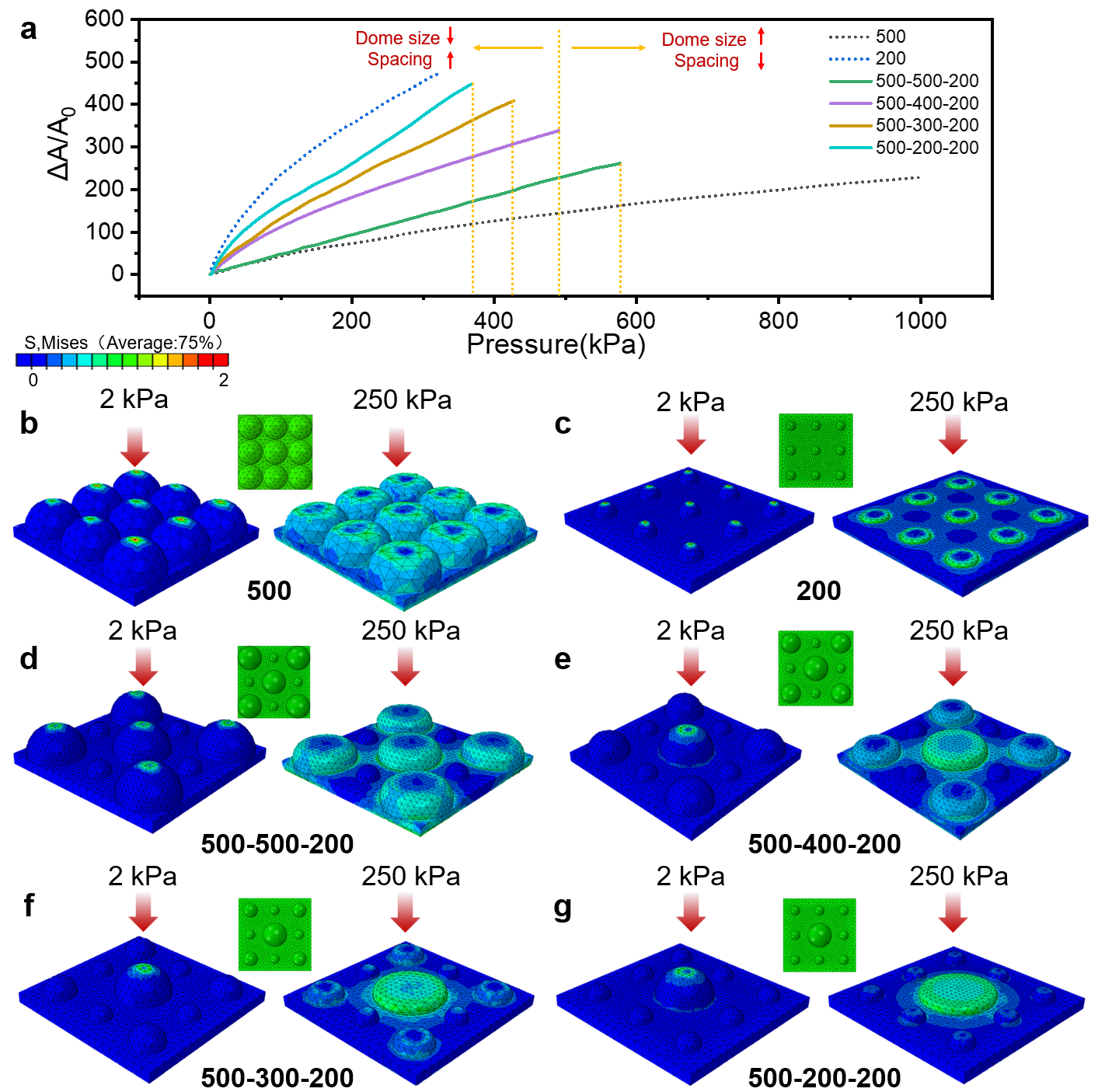


**Figure S9.** **Finite element analysis of dome structures with different dimensions and configurations.** (a) Relationship between compression and pressure load of dome structures with different dimensions. (b, c) Finite Element Analysis results of domes with T = 1 mm, D = 500 μm (b), D = 200 μm (c) at the loading pressure of 2 and 250 kPa, respectively. The resistance relative change rate is simplified to the area relative change rate, and the contact with the gear shaper electrode is not considered, which approximately reflects the law. (d, e, f, g) Finite Element Analysis results of domes with different dimensions and configurations at the loading pressure of 2 and 250 kPa. The diameter of domes in the fixed middle are D = 500 μm, and the diameter of domes in the middle of each side are D = 200 μm. Changing the diameter of domes located at the four corners to D = 500 μm (d), D = 400 μm (e), D = 300 μm (f), D = 200 μm (g).

Comparing the finite element analysis results of dome structures with diameters of 500 μm, 300 μm and 200 μm in Figure S8a, it can be found that smaller domes have higher sensitivity, but the compression range is reduced, which is consistent with the experimental results in Figure 3g. In order to explore the influence of different size domes on sensing performance in multistage structure, finite element analysis was carried out for domes with four different size combinations: 500-500-200, 500-400-200, 500-300-200, 500-200-200 (The configurations of these combinations are shown in Figure S8b-h). The results show that with the introduction of multistage structure, the sensor unit can combine the advantages of high sensitivity of small-size domes and large pressure range of large-size domes to achieve high sensitivity and large sensitivity range. The sensing curve of the multistage structure lies between the sensing performance curve of the unipolar structure of its combined size. In terms of sensitivity, 500-500-200 < 500-400-200 < 500-300-200 < 500-200-200, while the linear range is 500-500-200 > 500-400-200 > 500-300-200 > 500-200-200. Therefore, to obtain a larger linear range, more large-size dome structures can be introduced into the multistage structure, while more small-size dome structures can be added to the multistage structure to obtain higher sensitivity.

In addition, the distance between the domes also has a certain influence on the sensing performance. In the same area, greater spacing means less dome structure, so the structure is more prone to deformation, with a greater rate of change in the contact area, that is, sensitivity. This is confirmed by the experimental results in Figure 3h. In the contact process of the multistage structure, only the large dome is under pressure at the stage of low pressure. The change of the sensing curve of 500 (with nine 500 μm domes), 500-500-200 (with five 500 μm domes) and 500-200-200 (with one 500 μm dome) at the initial stage can reflect the influence of different spacing between domes on the sensing performance. It was found that 500-200-200 (with one 500 μm dome) had the greatest sensitivity, and 500 (with nine 500 μm domes) had the least sensitivity. This is also consistent with the experimental results in Figure 3h.

To provide an overview of how size changes and configurations affect sensing performance, the finite element analysis using the simulation model established here was carried out. On the one hand, the establishment of the simulation model can help us better understand the physical behavior of the multistage structure dome during the compression process, and provide a theoretical basis for the experimental results. On the other hand, the simulation can guide us to customize the sensing performance of the sensor in practical applications. The multi-stage dome structure simulation model constructed in this paper can be used as a bridge between theory and experiment, and provide theoretical basis and guidance for optimizing sensing performance.


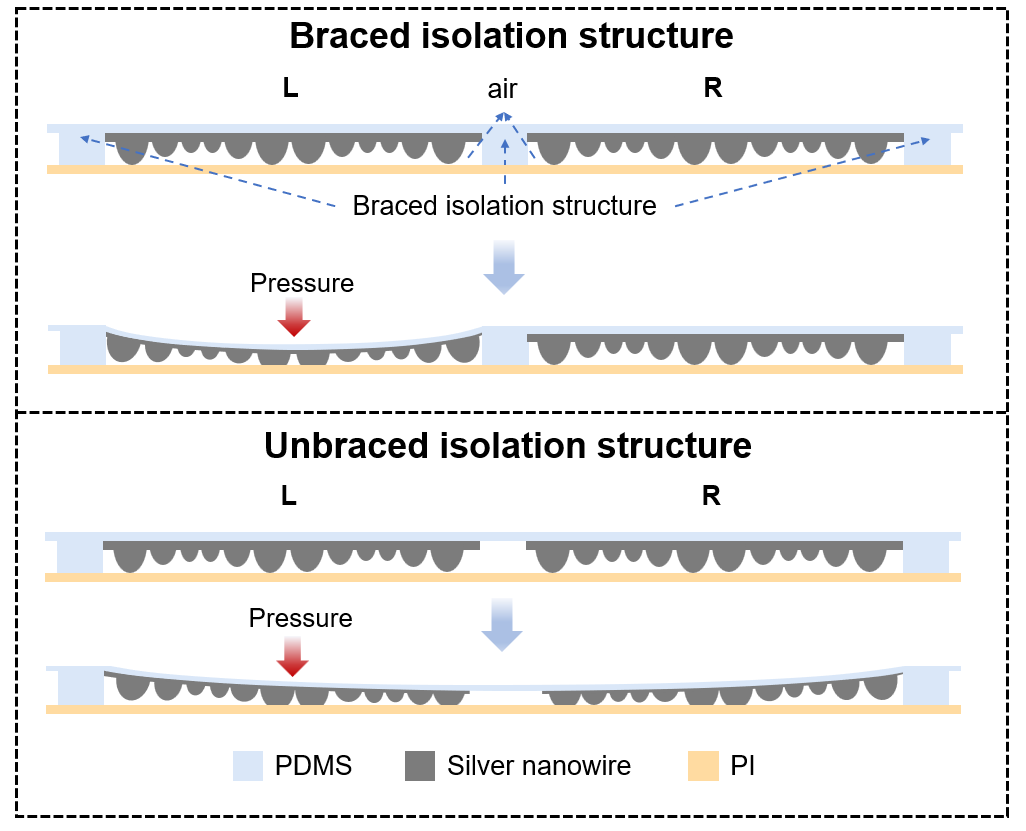


**Figure S10. Diagram of braced isolation structure and unbraced isolation structure.** A columnar supporting isolation structure is introduced between the sensing units of the sensor array. When external pressure *F* is applied to the working sensing unit, the limiting effect of the supporting isolation structure can effectively reduce the material deformation between adjacent sensing units. As a result, material deformations between sensing units can be isolated, eliminating the effect of signal crosstalk.


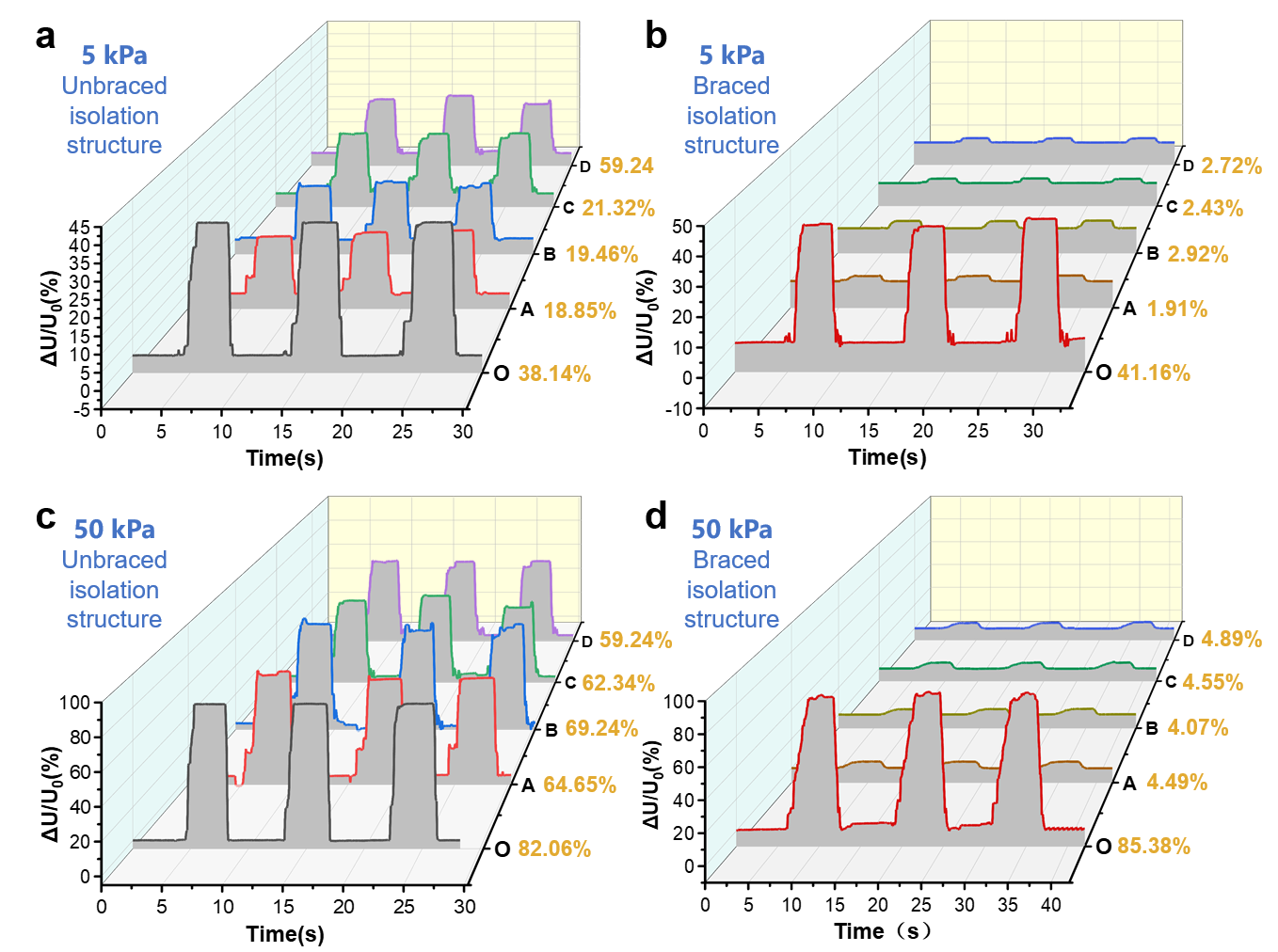


**Figure S11. Signal crosstalk test.** Pressure loads of 5 kPa and 50 kPa are applied successively against the central sensing unit O, and the process is repeated three times. Under each pressure load, the voltage change of the central sensing unit O and the surrounding sensing unit A, B, C, D is recorded. (a) Signal crosstalk test at 5 kPa pressure with sensor array with unbraced isolation structure. (b) Signal crosstalk test at 5kPa pressure in sensor array with braced isolation structure. (c) Signal crosstalk test at 50 kPa pressure with sensor array with unbraced isolation structure. (d) Signal crosstalk test at 50 kPa pressure in sensor array with braced isolation structure.

The formula for the voltage change rate is $\frac{\text{Δ}\text{U}}{\text{U}_{\text{0}}}$ (Figure S9).

The formula for calculating the crosstalk coefficient is:

$$\begin{aligned} \text{CTK}\text{=}\text{2}\text{0}\text{log}_{\text{10}} \frac{\text{R}_{\text{cen}\text{1}}\text{-}\text{R}_{\text{cen}\text{0}}}{\text{R}_{\text{s}\text{ur}\text{1}}\text{-}\text{R}_{\text{sur}\text{0}}}\text{=}\text{2}\text{0}\text{log}_{\text{10}} \frac{{\text{∆}\text{R}}_{\text{cen}}}{{\text{∆}\text{R}}_{\text{sur}}}\#\left( S18. \right) \end{aligned}$$

*CTK* is the calculated crosstalk coefficient (dB); $\text{R}_{\text{sur}\text{1}}$ is the resistance value of the surrounding sensing unit under pressure (Ω); $\text{R}_{\text{sur}\text{0}}$ is the resistance value of the surrounding sensing unit in the initial state (Ω); $\text{R}_{\text{cen}\text{1}}$ is the resistance value of the central sensing unit under pressure (Ω); $\text{R}_{\text{cen}\text{0}}$ is the resistance value of the central sensing unit in the initial state (Ω); ${\text{∆}\text{R}}_{\text{sur}}$ is the resistance change of the surrounding sensing unit before and after compression (Ω); ${\text{∆}\text{R}}_{\text{cen}}$ is the resistance change of the central sensing unit before and after compression (Ω).

The formula for calculating the array dispersion coefficient CV is:

$$\begin{aligned} \text{CV}\text{=}\frac{\text{σ}}{\overline{\text{y}}}\text{×100\%}\#\left( S19. \right) \end{aligned}$$

$\text{CV}$ is the dispersion coefficient, $\text{σ}$ is the standard deviation of the pressure response of each sensing unit, and $\overline{\text{y}}$ is the mean value of the pressure response of each sensing unit.


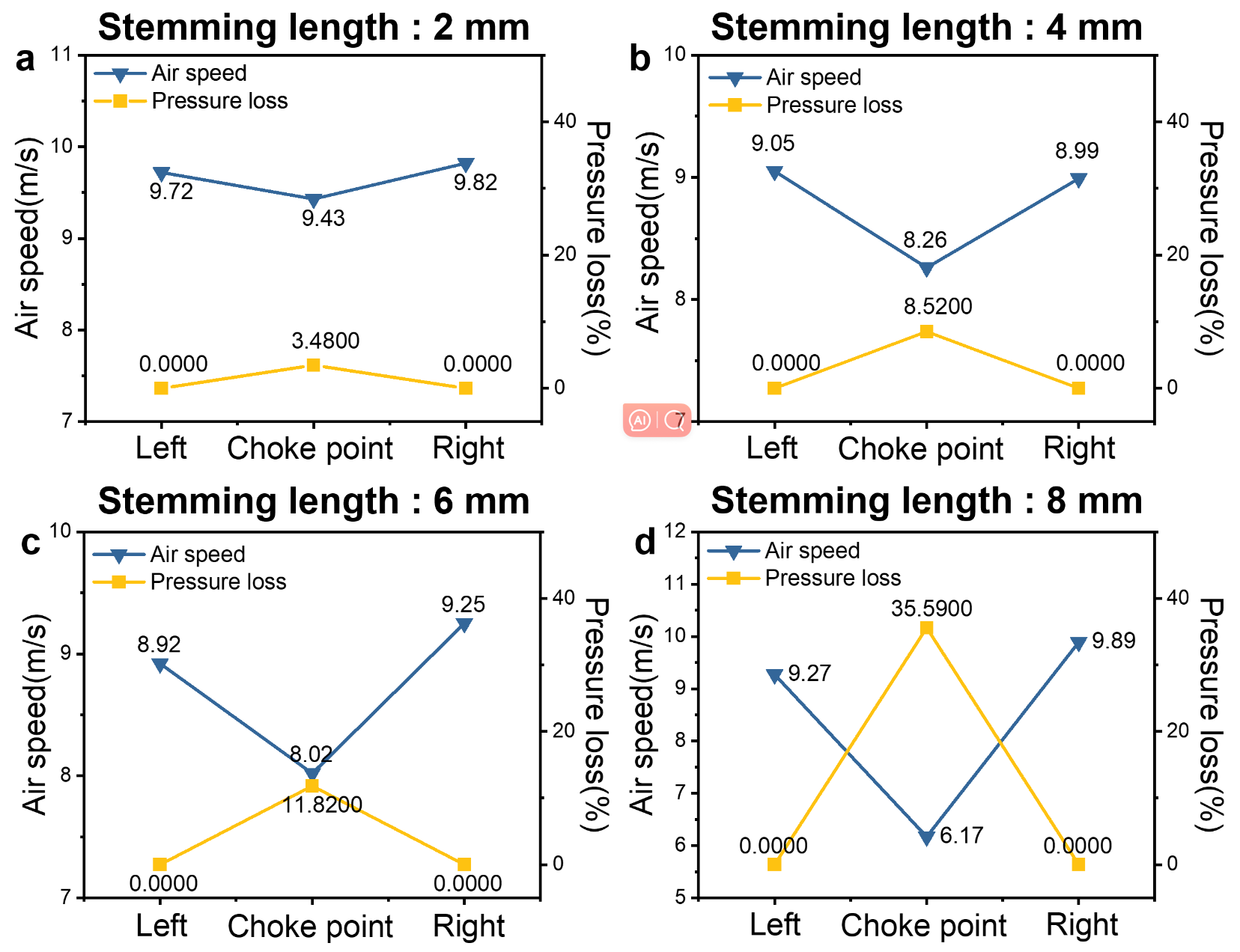


**Figure S12. The air speed and pressure loss of air knife under different plugging area lengths.** The length of clogging area has obvious influence on the cleaning effect of air knife. The flow rate loss is relatively small in the 2 mm, 4 mm and 6 mm clogging length range, but increases sharply as the length of the clogging area increases above 6 mm, and the glass cleaning quality will be significantly affected.


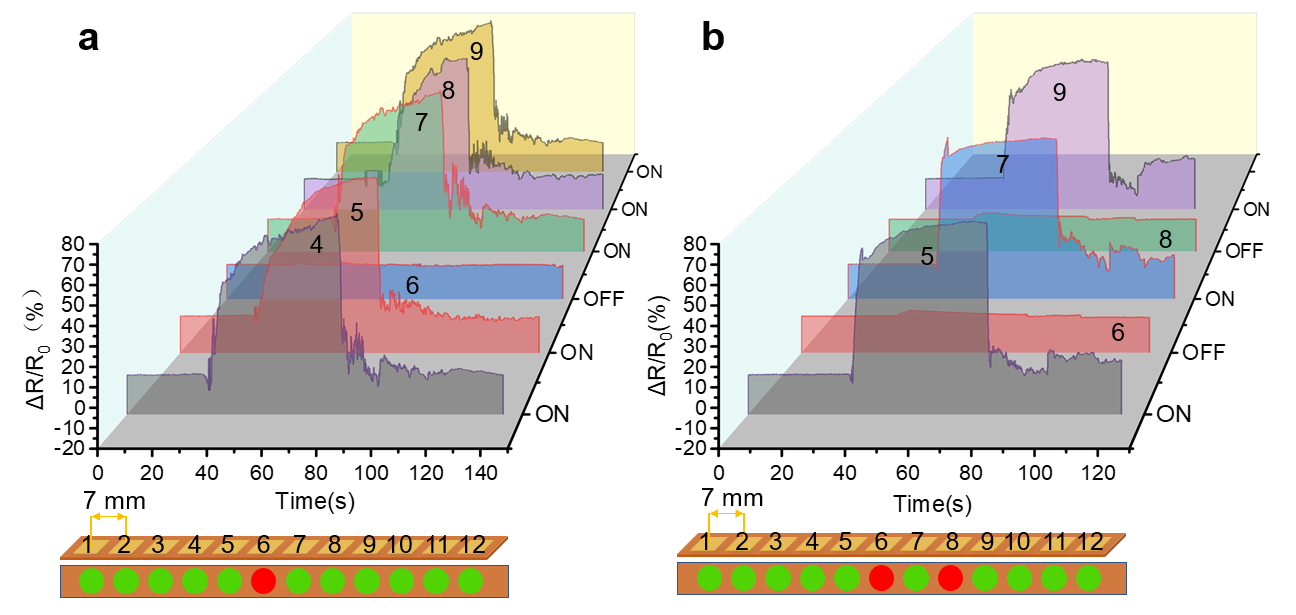


**Figure S13. Blockage location detection for air knives.** (a) The length of the blocked area is 7 mm. (b) The length of the blocked area is 14 mm.


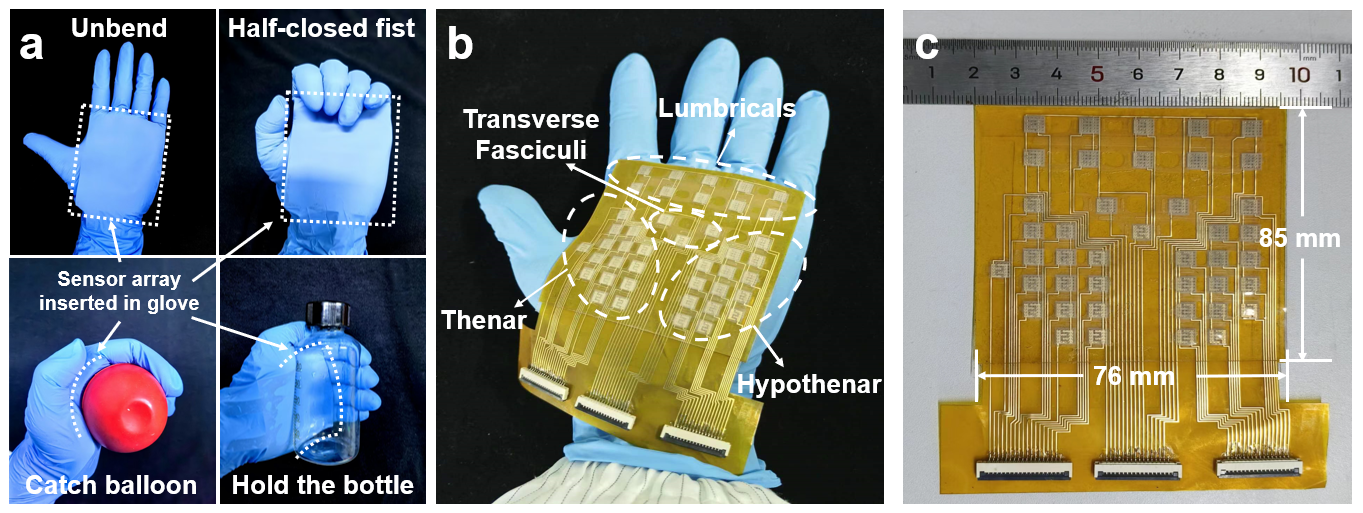


**Figure S14. Custom manufactured flexible pressure sensing array based on human palm muscle distribution.** (a) Sensor array inserted in glove for better sensing performance in the specific using process. In the process of various palm movements, the sensor array can always be closely attached to the palm surface to maintain good contact between the sensor unit and the palm surface to achieve high-precision measurement and reliability feedback. (b) Schematic diagram of the corresponding relationship between palmar muscle and Sensor unit distribution. (c) The size of the sensor array is designed to be 85 mm × 76 mm (4 mm × 4 mm for each sensor).

**Figure S15. Cloud image of pressure sensitive film thickness.** The thickness of the pressure-sensitive film obtained by moulding shows high uniformity at each point. The thickest T_max_ = 610 μm, the thinnest T_min_ = 590 μm, thickness error *δ* = Δ *T* / *T* × 100 % = 3.2% < 5%.

**Table S1.** Comparison between this work and previously reported pressure sensors

| **Artical** | **Sensitivity**  **(kPa^-1^)** | **Cycle stability**  **(times)** | **Array** | **Crosstalk resistance(dB)** | **Array consistency**  **(×10^2^)** |
| --- | --- | --- | --- | --- | --- |
| This work | 0.29 | 22000 | 12×12 | 26.62 | 1/9.62 |
| ACS Nano^[1]^ | 0.01 | 10000 | 8×8 | 6.6 | - |
| Small^[2]^ | 0.29 | 10000 | 4×8 | - | - |
| Adv. Funct. Mater.^[3]^ | 0.17 | 20 | 2×2 | - | - |
| Adv. Electron. Mater.^[4]^ | 0.06 | 500 | 6×6 | - | - |
| Adv. Mater.^[5]^ | 0.76 | 8000 | 4×5 | - | 1/18 |
| Adv. Sci.^[6]^ | 0.19 | 10000 | 10×10 | 47.24 | - |
| Adv. Mater. Technol.^[7]^ | 0.562 | 10000 | 3×3 | - | - |
| J. Colloid Interface Sci.^[8]^ | 2.6 | 600 | 6×6 | - | - |
| J. Mater. Chem. C^[9]^ | 2.09 | 500 | 1×1 | - | - |

While most sensor arrays do not consider array consistency between cells, our approach emphasizes high consistency manufacturing and fully considers the main performance metrics of high consistency sensing. Bao et al. proposed an array of highly sensitive pressure sensors entirely made of biodegradable materials, and the standard deviations associated with pressure sensitivities is 0.76 ± 0.14 kPa^−1^.^[5]^ By substituting the consistency calculation formula we proposed, the array dispersion coefficient CV can be obtained as 18%. In our work, the manufacturing process of the flexible pressure sensor array that can be molded with multi-pixel and high precision ensures the uniformity of the sensor unit performance and improves the consistency of the sensor array (array dispersion coefficient CV = 9.62%).

Pressure sensor arrays suffer from high crosstalk, resulting in inaccurate sensing, which consequently limits their use in such advanced applications. While most research do not pay attention to the problem of array crosstalk, only a few research calculated and compared the crosstalk coefficient of the pressure array. Park et al. fabricated large-area, flexible Molybdenum disulfide (MoS_2_) tactile sensors driven by an active-matrix (AM) backplane circuitry.^[1]^ The integrated MoS2-based active-matrix potentially eliminated the crosstalk issue, effectively increases the crosstalk isolation value to 24.8 dB. Luo et al. fixed a sensing membrane onto a high-stiffness substrate with cavities, forming a stable braced isolation to provide an excellent crosstalk-free capability.^[6]^ In our work, the braced isolation structure is proposed to effectively improve the crosstalk resistance performance of the sensor array (crosstalk coefficient: 26.62 dB). The braced isolation structure reduces the strain transfer between the sensor units, limiting the mechanical deformation caused by strain transfer, and thus inhibits the signal crosstalk phenomenon between the sensor arrays.

Since the conductive layer of resistive sensors is primarily produced through direct adhesion, infiltration, deposition, and sputtering processes, the inconsistency in materials between the microstructure layer and the conductive layer results in insufficient bonding strength between the two layers. The conductive layer may fall off when it is in service for a long time, thus affecting the working stability of the sensor. Chen et al. use the optimized graphene films to produce flexible transparent pressure sensor arrays based on a cavity design.^[3]^ However, due to limitation of the material, the sensor array can only achieve stable output for 20 cycles. Pyo et al. present a capacitive tactile sensor comprised of monolayer graphene electrodes that are separated by spacers which form air gaps and achieve high reliability against repeated deformation of 500 cycles at a bending radius of 8 mm is demonstrated.^[4]^ In our work, the insertion of the silver nanowire conductive layer into the polydimethylsiloxane (PDMS) microstructure layer to form the embedded sensing layer solves the problem of the shedding of the conductive layer in long-time use and achieves high cycle stability (22000 times loaded at 15 kPa pressure).

Some randomly sized protrusions created through the sandpaper ^[7]^, lotus leaf ^[9]^ and rose ^[8]^ were reported to achieve high sensitivity in large linear range. Similar to our work, such structures also achieve high sensitivity and large linear range through protrusion structures of different sizes, however, the surface of these structures is random and cannot be regulated, resulting in the sensing performance of sensors manufactured by these methods is not adjustable and has a certain randomness. The random and non-reproducible structure and performance strongly hamper the expansion and practical applications of the sensors. By contrast, our proposed biomimetic regular structure can be expanded to multi-pixel sensor array with high consistency through large-scale high precision imprinting technologies.

In addition, due to the limitation of materials and manufacturing technology, the existing flexible pressure sensing arrays are difficult to manufacture in multi-pixel. Wang et al. proposed a method for fabricating gel pressure sensors using a hydrophobic/hydrophilic patterned surface and developed a 4 × 8 array of conductive gel droplets.^[2]^ In our work, a reproducible high-precision imprinting technology is proposed to achieve high consistency expansion of the sensing unit to multi-pixel sensor array (12×12 Pixels within 300 mm × 300 mm area).

In summary, our proposed multistage dome sensing array has these advantages: 1) Using the step by step contact sensing principle of multistage structure, the sensitivity of large pressure ranges can be improved. The biomimetic multistage dome structure is pressurized to gradually contact the electrode surface, breaking through the limitations of maintaining high sensitivity under large sensitive range; 2) The conductive material is embedded into the microdome structures in imprinting process to improve the stability and the high precision imprinting process can solve the problem of poor consistency in each unit in the large-area expansion of multi-pixel array; 3) The braced isolation structure effectively improves the crosstalk resistance performance of the sensor array. The braced isolation structure reduces the strain transfer between the sensor units, limiting the mechanical deformation caused by strain transfer, and thus inhibits the signal crosstalk phenomenon between the sensor arrays; 4) The manufacturing process of the flexible pressure sensor array that can be molded with multi-pixel and high precision ensures the uniformity of the sensor unit performance and improves the consistency of the sensor array.

REFERENCES

[1] Y. J. Park, B. K. Sharma, S. M. Shinde, M. S. Kim, B. Jang, J. H. Kim, J. H. Ahn, *ACS Nano* **2019**, 13, 3023.

[2] Z. Wang, Q. Cai, L. Lu, P. A. Levkin, *Small* **2024**, 20, No. 2305214.

[3] Z. Chen, T. Ming, M. M. Goulamaly, H. M. Yao, D. Nezich, M. Hempel, M. Hofmann, J. Kong, *Adv. Funct. Mater.* **2016**, 26, 5061.

[4] S. Pyo, J. Choi, J. Kim, *Adv. Electron. Mater.* **2018**, 4, 1700427.

[5] C. M. Boutry, A. Nguyen, Q. O. Lawal, A. Chortos, S. Rondeau-Gagné, Z. Bao, *Adv. Mater.* **2015**, 27, 6954.

[6] H. Luo, X. Chen, S. Li, J. Xu, X. Li, H. Tian, C. Wang, B. Li, M. Zhang, B. Sun, J. He, J. Shao, *Adv. Sci.* **2024**, 11, 2403645.

[7] Y. Gao, G. Yu, T. Shu, Y. Chen, W. Yang, Y. Liu, J. Long, W. Xiong, F. Xuan, *Adv. Mater. Technol.* **2019**, 4, 1900504.

[8] B. Chen, L. Zhang, H. Li, X. Lai, X. Zeng, *J. Colloid Interface Sci.* **2022**, 617, 478.

[9] K. Nie, Z. Wang, H. Zhou, R. Tang, X. Shen, Q. Sun, *J. Mater. Chem. C* **2020**, 8, 16113.
